# Body weight index indicates the responses of the fecal microbiota, metabolome and proteome to beef/chicken-based diet alterations in Chinese volunteers

**DOI:** 10.1038/s41522-022-00319-7

**Published:** 2022-07-12

**Authors:** Di Zhao, Kai Shan, Yunting Xie, Guanghong Zhang, Qi An, Xiaobo Yu, Guanghong Zhou, Chunbao Li

**Affiliations:** Key Laboratory of Meat Processing, MOA, Key Laboratory of Meat Processing and Quality Control, MOE, Jiang Synergetic Innovation Center of Meat Production, Processing and Quality Control, College of Food Science and Technology, Nanjing Agricultural University, 210095 Nanjing, P. R. China

**Keywords:** Microbiome, Microbial ecology

## Abstract

Relationships between meat consumption and gut diseases have been debated for decades, and the gut microbiota plays an important role in this interplay. It was speculated that the gut microbiota and relevant indicators of hosts with different body weight indexes (BMIs) might respond differentially to meat-based diet alterations, since lean and obese hosts have different gut microbiota composition. Forty-five young Chinese volunteers were recruited and assigned to high-, middle- and low-BMI groups. All of the volunteers were given a beef-based diet for 2 weeks and subsequently with a chicken-based diet for another 2 weeks. Body weight and blood indexes were measured, and fecal samples were obtained for 16S rRNA sequencing, metabolome and proteome analyses. The fecal metabolites of the low-BMI volunteers showed greater sensitivity to meat-based diet alterations. In contrast, the fecal proteome profiles and blood indexes of the high- and middle-BMI volunteers indicated greater sensitivity to meat-based diet alterations. Replacing the beef-based diet with the chicken-based diet largely changed operational taxonomic units of *Bacteroides* genus, and thus probably induced downregulation of immunoglobulins in feces. Compared with the beef-based diet, the chicken-based diet decreased inflammation-related blood indexes, especially in high- and middle-BMI volunteers. This work highlighted the role of BMI as an important factor predicting changes in gut homeostasis in response to meat consumption. Compared with the chicken-based diet, the beef-based diet may induce more allergic and inflammation-related responses in high- and middle- BMI Chinese at the current level.

## Introduction

The gut microbiota has been shown to be associated with many physiological activities^1,2^. Diet, medication, geographic origin, genetics and age have been revealed to change the gut microbiota composition^3^. In particular, dietary components are critical factors that regulate gut microbiota composition and functions^4^. The gut microbiota, in turn, affects the absorption and metabolism of dietary components and further profoundly affects host physiology through the gut-liver axis, the gut-brain axis and other pathways^5–7^. Carbohydrates are mainly degraded by the gut microbiota into short-chain fatty acids (SCFAs), which serve as energy sources and immunological regulators for the hosts^8^. High-fat diets have been reported to induce gut dysbiosis and metabolic disorders through lipopolysaccharide-mediated pathways^9^. Dietary proteins can be transformed by the gut microbiota into SCFAs, branched-chain fatty acids, phenyl propionate, p-cresol, phenyl acetate, indole propionate, indole acetate and amines, some of which can negatively affect human health^10^.

Meat is an important source of protein, heme iron and B vitamins. The associations of red and processed meat consumption with human health have been debated for decades. Many epidemiological studies have reported that excessive intake of red/processed meat can cause colonic cancers, cardiovascular diseases and diabetes mellitus^11,12^. However, several recent studies have demonstrated that red meat consumption at the current levels might have little or no effect on morbidity and mortality due to cardiometabolic diseases^13,14^. Our animal studies revealed that extracted meat proteins exerted both beneficial and adverse impacts on the gut microbiota composition and related physiological responses^15–18^. However, these conclusions obtained using extracted meat proteins cannot be extended to the whole meat, and the organs of mice and rats involved in food digestion and metabolism are not exactly the same as those of humans. Therefore, the effects of meat consumption on human gut microbiota composition and function remain to be elucidated. In addition, discrepancies in the gut microbiota between high- and low-BMI hosts have been reported extensively, and microbiome alterations in obesity have been observed after weight loss interventions^19,20^. Therefore, the gut microbiota of humans with different BMIs could respond differently to meat consumption. This study aimed to investigate the effects of short-term intake of beef- and chicken-based diets on body weight, blood count indexes (including hemoglobin, erythrocytes, leucocytes, lymphocytes, monocytes, neutrophils, eosinophils, basophils, giant immature cell, hematocrit, mean corpuscular volume, mean corpuscular hemoglobin, mean corpuscular hemoglobin concentration, red blood cell distribution width and platelet), blood pressure, triglyceride content, serum total cholesterol content in blood samples, and microbiota, metabolites and proteomics in fecal samples from high-, middle- and low-BMI volunteers. The findings provide new insights into the associations between meat consumption and gut homeostasis.

## Results

**Fig. 1:**
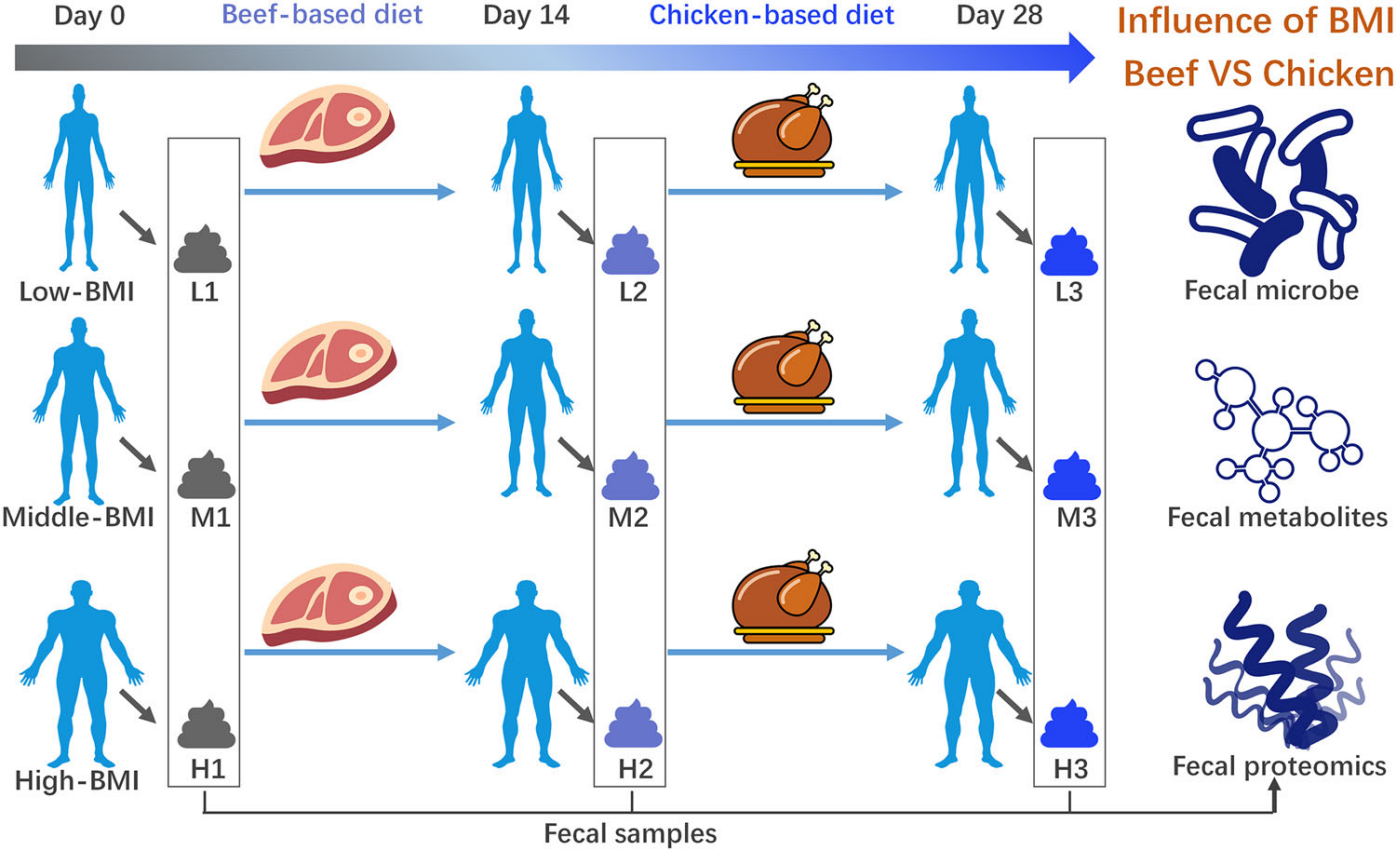
Schematic diagram of parallel assay to determine influence of meat-based diets on fecal microbes, metabolites and fecal proteomics of male volunteers with different BMI ranges. Volunteers in each BMI group were supplied with a beef-based diet for 2 weeks and then replaced by a chicken-based diet for another 2 weeks. The measured variables were compared among three BMI groups.

As shown in the CONSORT diagram in Supplementary Fig. 1, 45 Chinese male volunteers were recruited and assigned to three groups including high-BMI (BMI > 24), middle-BMI (24>BMI > 20), and low-BMI (BMI < 20) groups. They were supplied with the beef-based diets (Supplementary Table 1) for 2 weeks, and then were supplied with the chicken-based diets (Supplementary Table 2) for another 2 weeks. The feces and blood samples of all of the volunteers were collected before the diet management as day 0 samples, after 2 weeks of the beef-based diet as beef samples and after another 2 weeks of the chicken-based diet as chicken samples Fig. 1.

### Meat-based diet alterations induced an elevation in blood cholesterol and basophils but a reduction in monocytes, erythrocytes, hematocrit and platelet distribution width in high-BMI volunteers

Before meat-based diet alterations (Supplementary Tables 1 and 2), a survey regarding the dietary habits of 45 volunteers (Supplementary Table 3) was conducted, and the results are shown in Supplementary Fig. 2. All of the volunteers showed a similar frequency of physical exercise independent of BMI values (Supplementary Fig. 2A). The high-BMI volunteers had the highest frequencies of meat consumption, and over 50% volunteers consumed meat more than three times a week in all groups (Supplementary Fig. 2B). Chicken (41.3%) was the most commonly consumed meat in high-BMI volunteers, which was followed by pork, beef, mutton, fish and duck in a descending order (Supplementary Fig. 2C). By contrast, middle and low-BMI volunteers consumed more pork (48.0–52.5%) than chicken (17.5–29.6%) in daily diets, and less beef was consumed in the habitual dietary intake of middle- and low-BMI volunteers than in high-BMI volunteers (8.7%) (Supplementary Fig. 2C).

In Table 1, the triglyceride levels were elevated (*P* > 0.05) in the high-BMI volunteers after the chicken-based diet, whereas they remained constant in the middle- and low-BMI volunteers. In addition, in the high-BMI volunteers, the levels of mean corpuscular hemoglobin increased during the diet alterations (*P* < 0.05), whereas the values of monocytes, erythrocytes, hematocrit and platelet distribution width decreased (*P* < 0.05) when the beef-based diet was replaced by the chicken-based diet. In contrast, only 2 and 1 items showed significant changes in middle-BM (basophils and mean corpuscular hemoglobin concentration) and low-BMI volunteers (basophils) when using the chicken-based diet to replace the beef-based diet. These results suggest that the blood indexes of volunteers with high BMI are more sensitive to meat-based diet alterations.

**Table 1:** Changes in the body weight and blood indexes induced by meat-based diets.

Indexes	H1	H2	H3	M1	M2	M3	L1	L2	L3
Body weight (kg)	66.4 ± 9.4	67.6 ± 9.7	67.3 ± 9.5	62.9 ± 6.5	63.5 ± 6.5	63.2 ± 5.9	64.9 ± 5.5	66.1 ± 5.5	66.1 ± 5.6
Systolic blood pressure (mm Hg)	129.7 ± 12.8	124.7 ± 11.6	123.7 ± 12.8	130.4 ± 9.0	130.9 ± 14.0	127.9 ± 10.5	127.2 ± 12.7	130.4 ± 15.5	128.3 ± 10.7
Diastolic blood pressure (mm Hg)	75.9 ± 12.6	73.2 ± 9.4	72.6 ± 9.3	75.7 ± 8.7	75.5 ± 7.5	74.5 ± 6.3	73.5 ± 5.8	74.2 ± 9.8	71.8 ± 9.3
Total cholesterol (mM)	4.6 ± 0.8^a^	3.9 ± 0.9^b^	4.2 ± 0.9^ab^	4.5 ± 0.9^a^	3.9 ± 0.7^b^	4.1 ± 0.9^ab^	4.0 ± 0.8	3.6 ± 0.9	3.6 ± 0.7
Triglyceride (mM)	1.0 ± 0.4^b^	1.2 ± 0.8^ab^	1.4 ± 0.6^a^	0.9 ± 0.4	1.1 ± 0.5	1.0 ± 0.4	0.8 ± 0.3	0.8 ± 0.4	0.8 ± 0.2
High density lipoprotein (mM)	1.3 ± 0.2	1.3 ± 0.3	1.2 ± 0.2	1.3 ± 0.3	1.3 ± 0.2	1.3 ± 0.2	1.2 ± 0.2	1.2 ± 0.2	1.3 ± 0.2
Low density lipoprotein (mM)	2.6 ± 0.6^a^	2.1 ± 0.5^b^	2.5 ± 0.8^ab^	2.5 ± 0.8^a^	2.0 ± 0.6^b^	2.3 ± 0.8^ab^	2.2 ± 0.6	2.0 ± 0.7	1.9 ± 0.7
Blood glucose (mM)	4.9 ± 0.2	4.7 ± 0.4	4.7 ± 0.4	4.8 ± 0.3	4.6 ± 0.3	4.8 ± 0.4	5.1 ± 0.4	4.9 ± 0.4	4.8 ± 0.6
Leucocytes (10^9^/L)	6.4 ± 1.3	6.0 ± 1.2	6.2 ± 1.3	5.8 ± 1.2	5.8 ± 1.1	6.5 ± 1.0	6.6 ± 1.0	6.6 ± 0.9	6.6 ± 1.5
Lymphocytes (%)	37.8 ± 8.9	38.8 ± 7.2	37.1 ± 8.2	39.2 ± 8.2	38.5 ± 7.0	38.3 ± 7.0	37.3 ± 6.0	36.4 ± 9.1	35.8 ± 10.5
Monocytes (%)	6.0 ± 1.5^b^	7.4 ± 2.2^a^	6.2 ± 1.2^b^	5.7 ± 1.1^b^	6.9 ± 1.4^a^	6.3 ± 1.3^ab^	6.4 ± 0.9^b^	7.4 ± 1.7^a^	6.8 ± 1.1^ab^
Neutrophils (%)	53.0 ± 9.3	50.0 ± 8.2	52.8 ± 8.4	50.4 ± 6.5	50.5 ± 6.7	51.9 ± 9.0	53.0 ± 6.1	52.5 ± 8.8	54.1 ± 10.8
Eosinophils (%)	2.3 ± 1.4	2.3 ± 1.7	2.5 ± 1.5	2.6 ± 1.7	2.5 ± 2.0	2.3 ± 1.9	2.5 ± 1.4	2.1 ± 1.0	2.3 ± 1.3
Basophils (%)	0.8 ± 0.2^b^	1.4 ± 0.5^a^	1.3 ± 0.6^a^	1.0 ± 0.3^b^	1.6 ± 0.3^a^	1.1 ± 0.4^b^	0.9 ± 0.1^b^	1.6 ± 0.4^a^	1.0 ± 0.4^b^
Giant immature cell (%)	0.3 ± 0.2	0.5 ± 0.2	0.5 ± 0.3	0.3 ± 0.1^b^	0.5 ± 0.2^a^	0.4 ± 1.7^ab^	0.4 ± 0.2	0.5 ± 0.3	0.4 ± 0.2
Erythrocytes (10^12^/L)	5.3 ± 0.3^a^	5.3 ± 0.2^a^	5.0 ± 0.4^b^	5.2 ± 0.3	5.2 ± 0.3	5.1 ± 0.3	5.3 ± 0.2	5.3 ± 0.2	5.2 ± 0.2
Hemoglobin (g/L)	159.6 ± 9.6	159.9 ± 9.2	156.5 ± 8.2	163.0 ± 9.3	160.9 ± 8.7	160.6 ± 9.8	159.9 ± 5.9	160.6 ± 6.8	160.3 ± 4.7
Hematocrit	0.46 ± 0.03^a^	0.46 ± 0.02^a^	0.43 ± 0.02^b^	0.47 ± 0.02^a^	0.46 ± 0.02^ab^	0.44 ± 0.02^b^	0.46 ± 0.02	0.45 ± 0.02	0.45 ± 0.02
Mean corpuscular volume (fL)	86.7 ± 2.7	86.2 ± 2.7	85.5 ± 3.0	88.7 ± 2.9	88.0 ± 3.0	87.3 ± 3.2	87.1 ± 2.2	85.6 ± 2.3	85.2 ± 2.3
Mean corpuscular hemoglobin (pg)	30.2 ± 1.3	30.1 ± 1.1	30.8 ± 1.6	31.1 ± 1.0	31.0 ± 0.9	31.5 ± 1.0	30.3 ± 0.7	30.4 ± 0.7	30.7 ± 1.2
Mean corpuscular hemoglobin concentration (g/L)	347.5 ± 5.9^b^	350.1 ± 4.9^b^	360.4 ± 13.3^a^	350.3 ± 4.3^b^	352.9 ± 4.5^b^	361.2 ± 10.0^a^	347.5 ± 4.0^b^	354.8 ± 4.8^a^	360.3 ± 9.9^a^
Red blood cell distribution width (%)	13.7 ± 0.6	13.4 ± 0.4	13.6 ± 0.4	13.2 ± 0.5	13.2 ± 0.4	13.3 ± 0.5	13.6 ± 0.4	13.4 ± 0.4	13.3 ± 0.4
Platelet (10^9^/L)	260.4 ± 41.8	254.2 ± 50.6	250.4 ± 43.8	230.8 ± 50.4	229.4 ± 59.0	228.1 ± 51.1	255.9 ± 54.8	252.2 ± 52.4	244.4 ± 46.4
Mean platelet volume (fL)	8.3 ± 0.7	8.9 ± 0.6	8.4 ± 0.7	8.7 ± 0.9^b^	9.3 ± 0.9^a^	9.0 ± 0.9^ab^	8.7 ± 1.1	9.3 ± 0.9	9.0 ± 1.0
Platelet distribution width (%)	13.4 ± 2.0^b^	14.7 ± 1.7^a^	13.5 ± 2.1^b^	14.8 ± 2.9	16.2 ± 3.0	15.1 ± 2.6	14.5 ± 3.3	15.7 ± 2.4	15.2 ± 2.8

^a^H1, M1 and L1 refer to high-, middle- and low-BMI groups before meat-based diet alterations, H2, M2 and L2 refer to each group after beef-based diet and H3, M3 and L3 refer to each group after chicken-based diet.

^b^The data were analyzed by two-way repeated measures ANOVA, and the means were compared by a Duncan’s post hoc test. Different superscript lowercases (a and b) denote significant differences induced by meat-based diet alteration in different BMI volunteers (*P* < 0.05).

### Fecal microbiota showed no significant changes at both phylum and genus level when the beef-based diet was replaced by the chicken-based diet

**Fig. 2:**
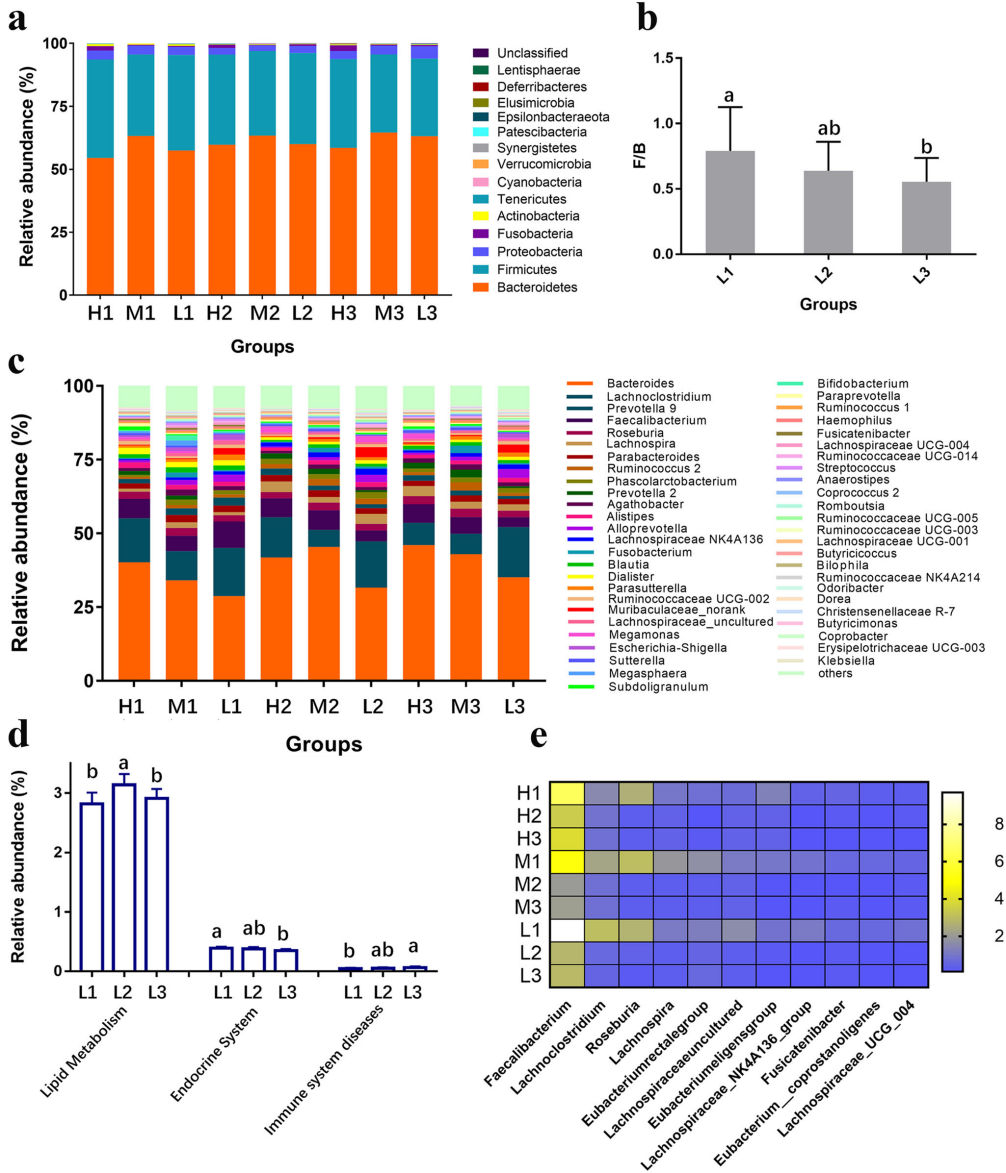
Changes in the composition and potential function of the fecal microbiota in response to meat-based diets or digests of meat proteins. **a** The microbiota at the phylum level, (**b**) the Firmicutes/Bacteroides (F/B) ratio in the low-BMI volunteers, (**c**) the microbiota composition at the genus level; **d** the differential functions of microbial genes; and (**e**) the differential genera in response to digests of meat proteins in vitro (the top 50 genera are shown), the color scale indicates the relative abundance (%) of genera. Error bars represent standard deviations.

Fecal microbiota composition and diversity indexes in response to meat-based diet alterations are shown in Fig. 2 and Supplementary Table 4. The relative abundance of the microbiota did not show significant differences among any of the volunteers at the phylum level (Fig. 2a). *Proteobacteria* appeared to be the phylum that was changed (*P* = 0.055) to the largest degree during the meat-based diet alteration. The *Firmicutes*/*Bacteroides* (F/B) ratios in all BMI volunteers were generally decreased after intake of the chicken-based diets, especially in the low-BMI volunteers (Fig. 2b).

The levels of fecal microbiota at the genus level in response to the meat-based diets are shown in Fig. 2c, but no genus was found to be significantly changed by replacing the beef-based diet with the chicken-based diet when the two-way repeated measures variance analysis and FDR correction were applied. Highest level of *Akkermansia muciniphila* was found in low-BMI volunteers, and a slight reduction was observed in the level of *Akkermansia muciniphila* when the beef-based diet was replaced by the chicken-based diet (Supplementary Table 5). Regardless of BMI factor, the beef-based diet was shown to increase the relative abundance of *Lachnospira*, the *Lachnospiraceae NK4A136 group* and *Ruminococcus 2* (Supplementary Table 6), whereas all genera didn’t show significant changes when using the chicken-based diet to replace beef-based diet. The Phylogenetic Investigation of Communities by Reconstruction of Unobserved States (PICRUSt) program was used to predict the functional alteration of gut microbiota, and the results are shown in Fig. 2d. Significant differences were only calculated in the low-BMI volunteers in the function of lipid metabolism (upregulated), endocrine-related function (downregulated) and immune system disease-related function (upregulated). When replacing the beef-based diet with chicken-based diet, only lipid metabolism was found to be significantly changed. These results suggest that the fecal microbiota in low-BMI volunteers was more sensitive to meat-based diet alteration.

Considering that protein is one of the most abundant nutrients in meat, the fecal microbiota was incubated in vitro with beef/chicken protein digests to verify whether protein is a crucial factor in meat affecting the gut microbiome. The differential genera (*P* < 0.05) of the top 50 (determined by abundance) fecal microbiota are listed in Fig. 2e and Supplementary Table 7. Digests of meat proteins generally decreased the relative abundances of *Faecalibacterium*, *Lachnoclostridium*, *Roseburia*, the *Eubacterium eligens group*, *Lachnospiraceae uncultured*, the *Lachnospiraceae NK4A136 group*, the *Eubacterium rectale group*, and *Eubacterium coprostanoligenes*. Eight genera in fecal samples from low-BMI volunteers were found to be significantly changed by meat protein digests, while only 3 genera in fecal samples of high-BMI volunteers showed significant changes. However, no genus was found to be significantly different between samples incubated with digests of beef protein and samples incubated with digests of chicken protein in all BMI groups.

### Species under *Bacteroidetes* was largely changed during the alteration from beef-based to chicken-based diets

**Fig. 3:**
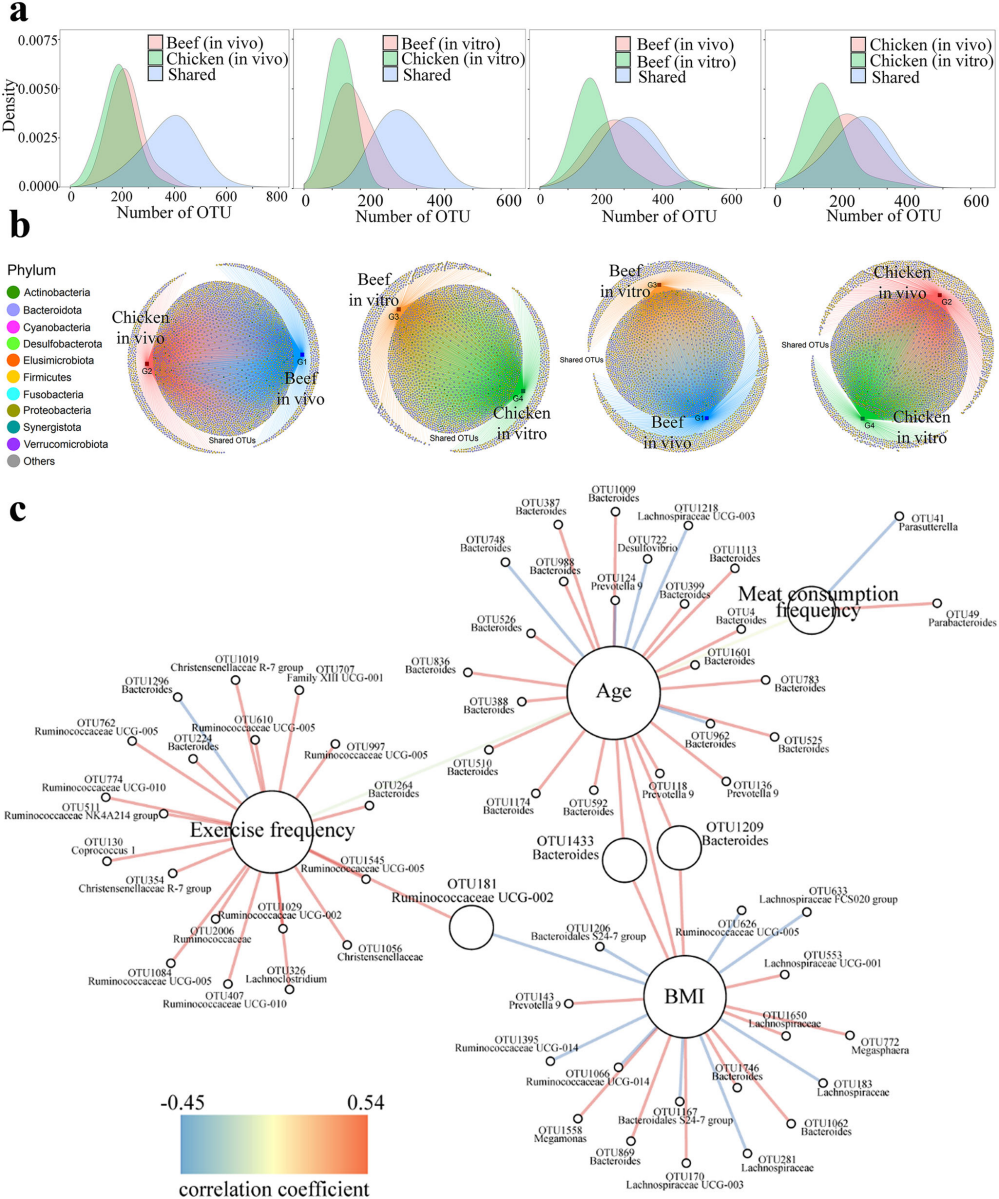
Comparison of the fecal microbiota in response to meat-based diet alteration or digests of meat proteins. **a** density distribution plot; **b** network plot at the phylum level; and (**c**) correlation analysis between volunteers’ indexes (age, exercise, BMI and meat consumption frequency) and fecal microbiota, the color scale indicates the Spearman correlation coefficients.

Figure 3a and b and relevant data in Supplementary Table 8 further show the differences in the fecal microbiota induced by meat-based diets. Fecal samples from volunteers after contained more operational taxonomic units (OTUs) after intake of the beef-based diet (595 per sample) than after intake of the chicken-based diet (560 per sample). Correspondingly, the cultures of fecal samples containing beef protein digests (522 OTUs per sample) had more OTUs than those containing chicken protein digests (475 OTUs per sample). Fecal samples from the volunteers who ate the beef-based diet had 240 unique OTUs belonging to the phylum *Bacteroidetes*, corresponding to 197 unique OTUs belonging to the same phylum in the fecal samples of volunteers who ate the chicken-based diet. Similarly, fecal samples incubated with beef protein digests had more unique OTUs belonging to the phylum *Bacteroidetes* than those incubated with chicken protein digests (333 OTUs vs. 179 OTUs). Thus, *Bacteroidetes* could be more adapted to the beef-based diet than to the chicken-based diet. At the OTU level, species under *Bacteroidetes* should be largely changed when replacing the beef-based diet by the chicken-based diet.

Correlations of fecal microbiota with age, exercise frequency, BMI and meat consumption frequency of volunteers are shown in Fig. 3c. Exercise frequency is shown to be positively correlated with the relative abundances of *Ruminococcaceae* (ten items) and *Christensenellaceae* (three items) species. BMI was found to be positively correlated with the relative abundances of *Lachnospiraceae* (five items) and *Bacteroides* (six items), but was negatively correlated with the relative abundance of *Ruminococcaceae* (3 items).

### Beef-based to chicken-based diet alterations induced greater changes of fecal metabolites in the low-BMI volunteers

**Fig. 4:**
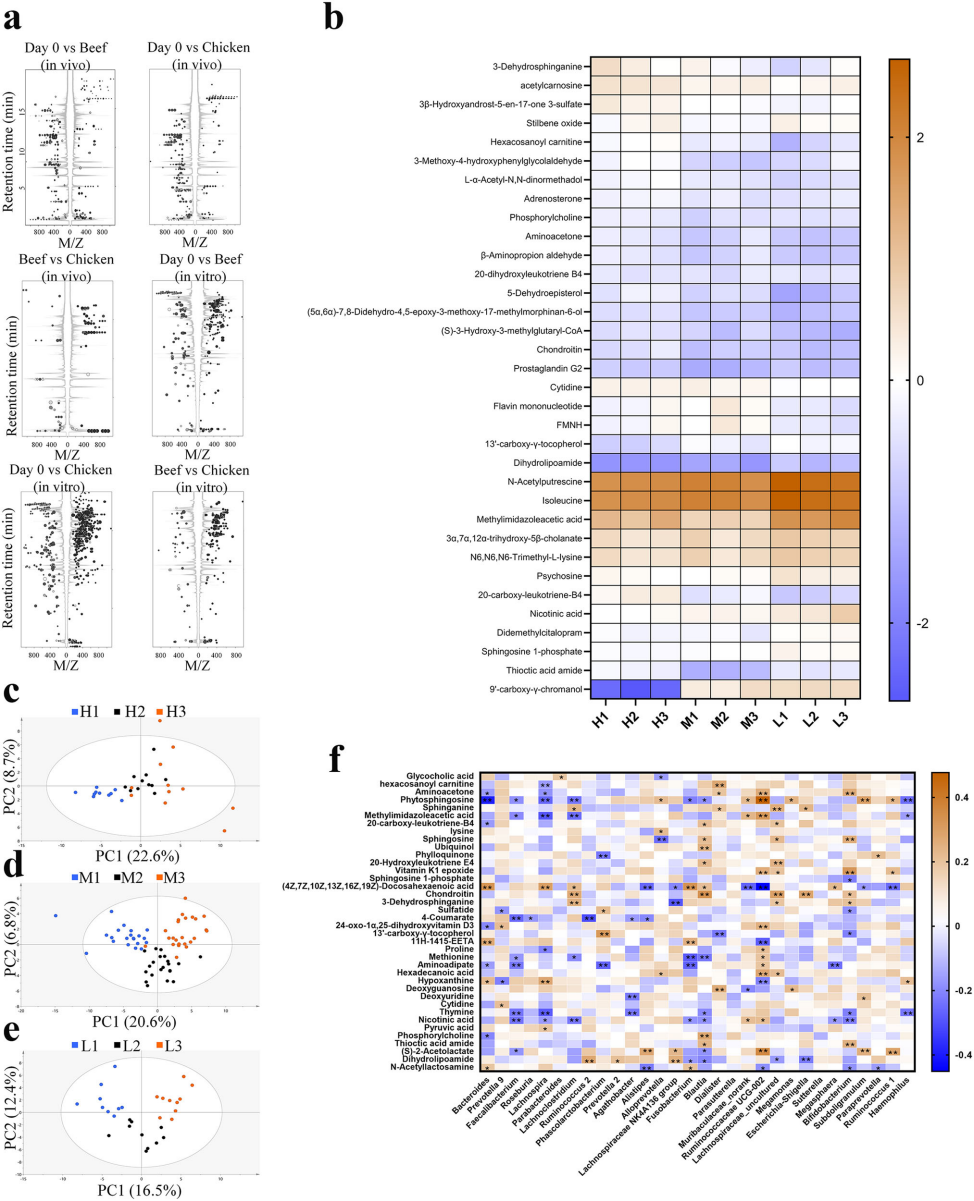
Comparisons of fecal metabolites in response to meat-based diet alteration in the high-, middle- and low-BMI volunteers. **a** Cloud map analysis of the differential m/z (*P* < 0.05); **b** Heatmap of fecal metabolites showed significant responses (*P* < 0.05) to meat-based diet alterations, the color bar indicates the relative content of metabolites; **c**–**e** PCA score plots of the fecal metabolites in high- (**c**), middle- (**d**) and low-BMI volunteers (**e**); **f** Heatmap for correlation coefficients between the fecal metabolites and the fecal microbiota. * and ** indicate that coefficients are significant (*P* < 0.05) and highly significant (*P* < 0.01), respectively; the color bar indicates the Pearson’s correlation coefficients.

There were 136 (beef vs. chicken) charge-to-mass ratio signals in mass spectra, which were significantly (*P* < 0.01) upregulated or downregulated in fecal samples of volunteers when the beef-based diet was replaced by the chicken-based diet (Fig. 4a). In the in vitro cultures, a total of 243 signals were different between samples incubated with beef protein digests and chicken protein digests (*P* < 0.01).

The metabolites in fecal samples of the high-, middle- and low-BMI volunteers are compared (Fig. 4b). Beef-based to chicken-based diet alterations increased the levels of prostaglandin G2, 3-methoxy-4-hydroxyphenyl acetaldehyde, nicotinic acid and N-acetylputrescine (*P* < 0.05). In Fig. 4b, the levels of 12, 9 and 6 metabolites were changed significantly in the fecal samples from the low-, middle- and high-BMI volunteers. In PCA plots, samples from low-BMI volunteers also show more obvious responses than those from the other volunteers (Fig. 4c–e). Therefore, fecal metabolites of low-BMI volunteers could be more sensitive to meat-based diet alterations. Regardless of BMI, 10 and 7 metabolites were down- and up-regulated when the beef-based diet was replaced by the chicken-based diet (Supplementary Table 9).

Correlation analysis between the metabolites and the fecal microbiota further indicated that 38 metabolites were significantly related to 30 genera of the fecal microbiota (*P* < 0.05, Fig. 4f). In particular, the relative abundances of *Ruminococcaceae UCG-002*, *Blautia*, *Bifidobacterium*, *Bacteroides* and *Lachnospira* were positively or negatively correlated with the levels of 15, 14, 11, 10 and 10 types of metabolites, respectively (*P* < 0.05). Positive correlation coefficients (r) were observed between phytosphingosine and *Ruminococcaceae UCG-002* (*r* = 0.48, *P* < 0.01), between (S)-2-acetolactate and *Ruminococcaceae UCG-002* (*r* = 0.35, *P* < 0.01), and between chondroitin and *Blautia* (*r* = 0.33, *P* < 0.01). Negative correlation coefficients were observed between docosahexaenoic acid and *Ruminococcaceae UCG-002* (*r* = −0.45, *P* < 0.01), between phytosphingosine and *Bacteroides* (*r* = −0.40, *P* < 0.01), and between coumarate and *Ruminococcus 2* (*r* = −0.32, *P* < 0.01).

### Beef-based to chicken-based diet alterations changed *Bacteroides*-related proteins and decreased hosts’ immunoglobulins in high- and middle-BMI volunteers

**Fig. 5:**
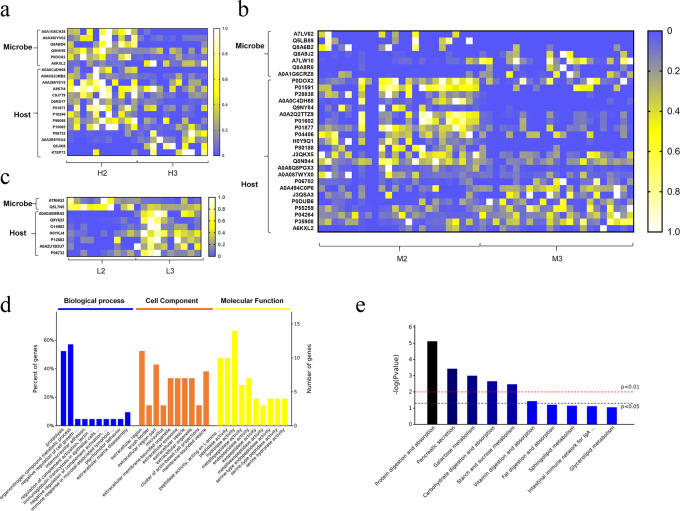
Differential fecal proteome profiling induced by diet alteration. **a**–**c** Heatmaps of metabolites from the high- (**a**), middle- (**b**) and low-BMI volunteers (**c**); **d**, **e** Functional annotation using GO (**d**) and KEGG orthology group assignments (**e**). The color bars in (**a**–**c**) indicate the relative abundance of identified proteins.

Profiling of differential fecal proteins from the host and microbiota is shown in Fig. 5. A greater number of differential proteins (*P* < 0.05) were identified in the fecal samples from high- (20 items) and middle-BMI volunteers (30 items) than in those from the low-BMI volunteers (only 9 items) after meat-based diet alterations (Fig. 5a–c). Compared with the proteins derived from the microbiota, more differential proteins were identified to be derived from the host. When the beef-based diet was replaced by the chicken-based diet, more host proteins were found to be significantly downregulated in high- (71.4%, Fig. 5a) and middle-BMI volunteers (65.2%, Fig. 5b). In contrast, all of the host proteins in Fig. 5c were significantly upregulated in low-BMI volunteers.

Notably, most differentially expressed microbial proteins in each group were derived from *Bacteroides*. In middle-BMI volunteers, A7LV62 (L-arabinose) and A7LW10 (phosphoenolpyruvate carboxykinase) were derived from *Bacteroides ovatus*. Q8A6B2 (glutamate dehydrogenase), Q8A9J2 (uronate isomerase) and Q8A8R0 (ferritin) were derived from *Bacteroides thetaiotaomicron*. Q5LB89 (Pyrophosphate--fructose 6-phosphate 1-phosphotransferase) was from *Bacteroides fragilis*, and A0A1G6CRZ8 (rubrerythrin) was from *Eubacterium oxidoreducens*. When the beef-based diet was replaced by the chicken-based diet, most of these *Bacteroides*-related proteins were downregulated in high- and low-BMI volunteers. These results are related to the changes in OTUs shown in Fig. 3b and Supplementary Table 8, suggesting that species under *Bacteroides* genus could be sensitive to the alteration from beef-based to chicken-based diets.

In host proteome, immunoglobulins in fecal samples from high- and middle-BMI volunteers were generally downregulated when the beef-based diet was replaced by the chicken-based diet (*P* < 0.05). In the feces of high-BMI volunteers, the levels of six immunoglobulins (A0A0C4DH68, A0A0G2JMB2, A0A286YEY5, D6RD17, P0DOX2, and P01871) decreased (*P* < 0.05) under the chicken-based diet. In the feces of middle-BMI volunteers, the level of seven immunoglobulins (A0A0C4DH68, A0A2Q2TTZ9, P01602, P0DOX2, P01591, A0A2Q2TTZ9, and P01877) decreased (*P* < 0.05) under the chicken-based diet. In contrast, no immunoglobulin was significantly affected in the feces of low-BMI volunteers during the diet alteration. In addition, in the feces from high- and middle-BMI volunteers, the levels of digestive enzymes were found to be decreased when replacing the beef-based diet by the chicken-based diet. The levels of anionic trypsinogen (Q5NV56) in feces of high-BMI volunteers, and aminopeptidase (P28838) and amylase in feces of middle-BMI volunteers after the beef-based diet were significantly higher than those after the chicken-based diet.

Regardless of BMI, the Gene Ontology (GO) and KEEG results of differential host proteins (chicken vs. beef) are shown in Fig. 5d and e. In GO analysis, differentially expressed proteins were mainly involved in proteolysis and organonitrogen compound metabolic processes in biological process function, and extracellular region and hydrolase activity were the most enriched functions in cell component and molecular function. In KEGG analysis, protein digestion and absorption, pancreatic secretion, galactose metabolism, carbohydrate digestion and absorption, starch and sucrose metabolism and vitamin digestion and absorption were the top 5 differentially enriched pathways. These results clearly indicated the different diet effects on protein digestion of the hosts between the chicken-based diet and the beef-based diet.

## Discussion

### BMI is an important factor indicating changes in gut homeostasis in response to meat consumption

Remodeling of the gut microenvironment by food has been widely studied, whereas the effect of host BMI on diet-induced remodeling of the gut microenvironment remains unclear^4^. In the present study, fecal microbiota and metabolites from low-BMI volunteers were found to be more sensitive to meat-based diet alterations. In contrast, fecal proteomics and blood indexes seemed more sensitive to the meat-based diet in the middle- and high-BMI volunteers. These results indicate that the diet effect on gut homeostasis could be dependent to some degree upon the BMI of hosts.

Food undergoes digestion, absorption and fermentation in the digestive tract and diet can immediately and reproducibly alter human gut microbiome^4^. Discrepancies in the gut microbiota between lean and obese people have been reported extensively, and higher F/B values have usually been observed in obese or high-BMI people^21^. The questionnaires revealed that the low-BMI volunteers had the lowest frequencies of meat consumption, and the high-BMI volunteers had the highest frequencies of meat consumption. This discrepancy in eating habits could also be partly responsible for the greater compromise of the fecal microbiota composition in the low-BMI volunteers to adapt to meat-based diet alterations. The differences in the metabolites between the beef- and chicken-diet groups could be due to the differences in the meat components, digestion processes by digestive enzymes and fermentation procedure by the microbiota^22^. Metabolites of the gut microbiota largely constitute metabolites in feces, which could largely account for the similar responses of fecal microbiota and metabolites to meat-based diet alterations^23^.

Meat-based diet alterations affected the blood indexes in more complex manners. Decreases in monocytes and basophils in low- and middle-BMI volunteers in response to the change from beef-based to the chicken-based diets might suggest a decrease in inflammation tendency^24,25^. Greater consumption of red meat has been reported to be significantly related to higher levels of inflammatory markers^26^. The relationship between obesity and chronic inflammation has been reported recently, and lean people usually have stronger anti-oxidative and anti-inflammatory systems compared with relatively obese ones^27–29^. Thus, it could account for the higher ability of low- and middle-BMI volunteers to reduce the inflammation tendency during diet alteration. Interestingly, fewer peptides were identified by proteomic analysis in the fecal samples of low-BMI volunteers, suggesting a higher degree of protein hydrolysis in the digestive tract, although more studies and evidence are still needed. Based on these results, host BMI should be a crucial factor when exploring the influence of diet on gut homeostasis.

### Beef-based diet induced greater inflammation-related and allergic responses than chicken-based diet

The influence of red meat on human health has been debated for decades. Beef, as a typical red meat, was compared with chicken regarding their influences on gut homeostasis. *Akkermansia muciniphila* has been inversely associated with obesity and inflammation, and negative correlation was found between the relative abundance of *Akkermansia muciniphila* and BMI under the Spearman correlation analysis (*r* = 0.37, *P* < 0.01) (Supplementary Fig. 3)^30,31^. After intake of the beef-based diet, the decreased level of *Akkermansia muciniphila* and elevated level inflammation-related blood cell counts (monocytes and basophils) in low- and middle-BMI volunteers confirmed these findings. In addition, an association between the heme level and the abundance elevation in *Akkermansia muciniphila* has been reported^32^, and beef has a higher level of heme that is embedded in myoglobin. Therefore, the higher level of *Akkermansia muciniphila* in fecal samples could be related to higher level of heme in the beef-based diet. However, the fecal microbiota composition remained relatively stable at the phylum and genus levels when the beef-based diet was replaced by the chicken-based diet. In addition to gut-microbiota-mediated bioprocesses, food composition, gastrointestinal digestion and absorption may also affect the metabolite composition in fecal samples. Chicken generally has a higher level of total purine and hypoxanthine than beef^33^, which may explain the higher level of hypoxanthine in the fecal samples after the chicken-based diet. Beef contains higher level of choline and carnitine, two precursors of TMA and TMAO, which have been related to increase cardiovascular risk and neurodegenerative disorders^34^. However, the levels of choline and carnitine in fecal samples were not significantly different between beef- and chicken-based diet groups, possibly due to a great variation existing among volunteers considering their different genetic background, life-style and absorption functions.

Notably, monocyte and basophil levels declined after replacement of the beef-based diet with the chicken-based diet, indicating a reduction in inflammatory-related responses. Recent studies have reported a relationship between N-glycolylneuraminic acid (Neu5Gc) and serum antibodies, promoting chronic inflammation^35^. Chicken has a lower level of Neu5Gc than beef^35^, which might partially explain the reduced monocyte and basophil levels in the chicken-based diet group. In line with this result, the levels of immunoglobulins in feces were decreased after replacing the beef-based diet by the chicken-based diet, which may also indicate the low level of inflammation-related and allergic reaction in digestive tract of volunteers after intake of the chicken-based diet since immunoglobulins are usually recruited when inflammation reactions occur^36^. The relationship between the immune system and microbiome is bi-directional, and the induction of a large shift in either will lead to a response in the other^37^. A recent study reported *Bacteroides ovatus* as a key species to trigger the production of intestinal immunoglobulin A^38^. Considering that the *Bacteroides ovatus-*related proteins were largely suppressed by the chicken-based diet, the lower level of immunoglobulins could be related to a reduction in *Bacteroides ovatus* abundance in the chicken-based diet group. In addition, beef proteins have been reported to be less digestible than chicken proteins^22^, hosts could have therefore secreted higher levels of digestive proteases. Some poor digested beef proteins could be potential antigen, which would also induce allergic and inflammation-related reactions.

### Chicken could be a better choice than beef for middle- and high-BMI Chinese

As suggested by the Nordic Nutrition Recommendations, the intake of red and processed meat should be limited to 500 g/week per capita. The World Cancer Research Fund (WCRF) suggests limitations of 200 g/week and 400 g/week for the consumption of red meat and poultry, respectively^39^. In this study, 1.3–1.5 kg/week of raw meat were cooked and consumed by volunteers. According to data released from the Organization of Economic Co-operation and Development in 2018, meat consumption in the USA, European Union and China was 2.43, 1.90 and 1.22 (similar to this work) kg/week, respectively. A recent review presented the idea of “less but better meat” in high-income countries because the overnourished population is increasing rapidly^39^. However, sufficient meat consumption remains very important in many low-income countries in which many people are at high risk of undernutrition. The present study confirms this viewpoint and suggests that it might be more appropriate for low-BMI Chinese to consume meat at the current level, but it could be better for high-BMI Chinese to reduce meat intake. In addition, compared with the beef-based diet, the chicken-based diet could be a better choice for Chinese at the current level, especially for high- and middle-BMI population. Chicken and beef are the second and third largest consumed meat in China, and beef consumption has increased greatly in recent years. Therefore, this work could provide a promising guide for meat consumption in China.

BMI is a key factor indicating responses of gut homeostasis to meat-based diet alterations in Chinese volunteers. The role of gut microbiota in precise nutrition has been discussed for many years, and BMI of hosts seems to be a crucial factor. Replacing the beef-based diet with the chicken-based diet, OTUs under *Bacteroides* genus were largely affected, leading to a reduction of immunoglobulins in feces, especially in high- and middle-BMI volunteers. In addition, the chicken-based diet also resulted in a reduction in inflammation-related blood indexes in high- and middle-BMI volunteers. Crossover assays with a larger number of volunteers are still needed, since the intra-group variations of volunteers could be greater than model animals considering the diversity of hosts’ genetic background and life-style.

## Methods

### Volunteer recruitment and diet alterations

In total, 45 male Chinese volunteers (Supplementary Table 3), aged 18–27 years old, were recruited from 150 candidates at Nanjing Agricultural University. All participants provided written informed consent to take part in the study. None of the participants had access to antibiotics within 3 months. Volunteers with similar body weight were assigned to one of three groups: high-BMI (11 volunteers, BMI > 24), middle-BMI (24 volunteers, 24>BMI > 20) and low-BMI (10 volunteers, BMI < 20). The study was approved by the Ethics Committee of Nanjing Agricultural University (SYXK < Jiangsu> 2011-0037). In addition, this study was registered in the clinical trial registration platform (ICTRP) (No. ChiCTR-ROC-17010926).

All of the volunteers were supplied with the beef-based diet (Supplementary Table S1) for 2 weeks, and then the chicken-based diet (Supplementary Table 2) for another 2 weeks. The feces and blood of all volunteers were collected before the first diet (day 0 samples), after 14 days of the beef-based diet (beef samples) and after another 14 days of the chicken-based diet (chicken samples).

### Blood index measurements

Blood pressure was measured by a HEM-90 automatic electronic sphygmomanometer (Hengfeng, Jiangsu, China). Blood count analyses were carried out on a 7180 automatic biochemical analyzer (Hitachi, Japan). Each sample was measured for three times to obtain an average.

### In vitro cultivation of fecal microbiota with beef or chicken protein digests

Beef and chicken were collected from the same source as the above diets. Beef and chicken proteins were isolated from beef longissimus dorsi muscle and chicken pectoralis major muscle obtained from a Sushi company (Jiangsu, China) (Supplementary Method 1)^16^. In vitro static digestion (Supplementary Method 2) was conducted to obtain beef and chicken protein digests^40^. The in vitro cultivation of fecal microbiota with beef and chicken protein digests was performed according to the procedures of Li et al.^41^ The fecal samples from each volunteer were collected in sterile sampling bags, and then transferred into a beaker that was bubbled continuously with N_2_. The feces were mixed in equal weight and homogenized. The homogenates were mixed with phosphate buffer saline to prepare slurry (10% (w/v)). The slurry was filtered through three layers of medical gauze and bubbled with N_2_ before use. The medium was prepared with 4 g/L beef or chicken protein digests, 40 mg/L K_2_HPO_4_, 40 mg/L KH_2_PO_4_, 10 mg/L MgSO_4_•7H_2_O, 10 mg/L CaCl_2_•6H_2_O, 0.1 g/L NaCl, 5 mg/L hemin, 30 g/L glucose, 2 mL /L Tween 80, 0.5 g/L bile salts, 0.5 mg/L vitamin K, 1 mg/L resazurin and 40 g/L NaHCO_3_. The medium pH was adjusted to 6.8 before sterilization. Then, the medium was bubbled with CO_2_ before cysteine (0.5 g/L) was added. In an AW200SG anaerobic box (Electrotek, United Kingdom), 9 mL of medium was mixed with 1 mL of the fecal slurry and incubated at 37 °C for 48 h. The culture was centrifuged at around 2000 × *g* for 3 min to obtain a bacterial suspension for sequencing.

### 16S rRNA sequencing of fecal microbiota

Microbiota in fecal samples or in vitro cultures was analyzed by 16S rRNA high-throughput sequencing. Fecal samples (2 g) were dissolved in 10 mL of phosphate buffer saline and homogenized at 4500 rpm for 3 min to obtain a bacterial suspension. DNA was collected from the suspended cultures or fecal samples using a QIAamp DNA stool mini kit (Qiagen, Strasse, Hilden, Germany). The DNA concentration was identified using agarose electrophoresis, and the DNA purity was identified by the value of OD260 nm/OD280 nm on a micro-spectrophotometer. DNA samples were diluted before further analysis. The forward and reverse primers were designed as 515 F (5′-GTGCCAGCMGCCGCGG-3′) and 907 R (5′-CCGTCAATTCMTTTRAGTTT-3′) to amplify the V4 hypervariable regions of the 16 S rDNA gene. PCR was conducted in a GeneAmp^®^9700 system (ABI, Foster City, CA, USA). The PCR products were identified by 2% agarose gel electrophoresis. Subsequently, the PCR products were subsequently recovered by a DNA recovery kit (Axygen, Foster City, CA, USA) and quantified using the Quantifluor^™^ system (Promega, Madison, WI, USA). The PCR products were measured in a MiSeq sequencing (Illumina, San Diego, CA, USA) using a 300 bp paired-end model. The paired-end reads were assembled in FLASH (https://ccb.jhu.edu/software/FLASH). Each sample was measured for one time.

After screening, filtering, and pre-clustering, gaps in each sequence were removed from all of the samples to reduce noise, OTUs were clustered with ≥97% similarity using UPARSE (http://drive5.com/uparse/), and chimeric sequences were identified using UCHIME (http://drive5.com/usearch/manual/uchime_algo.html). The affiliation of each sequence was analyzed by RDP Classifier (http://rdp.cme.msu.edu/) against the SILVA (http://www.arb-silva.de) data with a confidence threshold of 70%. Alpha- and β-diversity analyses were performed based on OTUs normalized by a standard of sequence number corresponding to the sample with the least sequence by performing the “single_rarefation.py” procedure in QIIME platform. PICRUSt program was used to predict the functional alteration of gut microbiota. The OTU data obtained were applied to generate BIOM files formatted as input for PICRUSt v1.1.09 with the make.biom script usable in the Mothur^42^. OTU abundances were mapped to Greengenes OTU IDs as input to speculate about the alteration of microbiota functions^42^. Age, BMI, frequency of exercise, frequency of meat consumption and relative microbial abundance were used for Spearman correlation analysis using MATLAB 2018a (The Math Works Inc., Natick, MA, USA). Variables with a correlation coefficient >0.35 and less than −0.35 with age, BMI, frequency of exercise, frequency of meat consumption or relative abundance of microorganisms were plotted using Cytoscape v 3.7.2.

### LC-MS/MS analysis to identify fecal metabolites

The LC-MS/MS method was used to separate and identify the metabolites^16^. Fecal samples (10 mg) were treated with 3 g of zirconium beads, and then mixed with 1 mL of methanol. The mixture was homogenized at 5000 rpm for 180 s at 4 °C and then homogenized again at 12000 rpm for 20 min. The supernatant (450 µL) was collected and then dried by nitrogen blowing. The dried sample was dissolved in 5 mL of formic acid solution (0.1%) and loaded in a TripleTOFTM 5600 LC-MS/MS (AB Sciex). A gradient elution was conducted in an XSelect CSH C18 column (2.5 μm × 2.1 × 150 mm, Waters) using 0.1% formic acid aqueous solution as eluent A and methanol as eluent B at a flow rate of 0.3 mL/min as follows: 0−1 min, 5% eluent B; 1−16 min, 5% -95% eluent B; 16–19 min, 95% eluent B; 19–22 min, 95% to 0% eluent B. A Q Exactive system (Thermo Scientific, Fremont, CA, USA) was used to detect the MS/MS in positive ion mode. Each sample was measured for one time.

Peakview 2.1 software (AB Sciex, Singapore) was used for peak alignment and convolution. Signals with poor kurtosis were removed for culling and screening. Markerview software (AB Sciex, Singapore) was then used for data normalization (Pareto scaling method), and the data were processed on an XCMS workstation (http://xcmsonline.scripps.edu) to identify the m/z. Then the m/z was searched against the ChemSpider database (http://www.chemspider.com/) and Metlin database (https://metlin.scripps.edu) in Markerview software to identify the potential metabolites. The cutoff of FDR for metabolites identification was set to 0.1. The correlations between different relative microbial abundance and metabolite intensity were performed by Pearson’s correlation analysis in SAS software, version 9.2 (SAS Institute Inc., NC, USA), and figures were constructed using GraphPad Prism (version 7.0.0; GraphPad Software, San Diego, CA).

### Fecal proteomic analysis

Proteins and digests derived from diets, host and gut microbiota in fecal samples were extracted using RIPA buffer, and the protein concentration was determined using a BCA kit (Thermo Scientific, Rockford, IL, USA). Protein samples (200 µg) were denatured, alkylated and digested by trypsin. The trypsin-treated samples (2 µg) were desalted using C18 ZipTip pipette tips and dissolved in 0.1% formic acid to obtain peptide solution (~4 µg/µL). Then the peptide samples were loaded into a Nano LC system (Thermo Fisher Scientific, Waltham, MA, USA) with an Acclaim PepMap RSLC C18 column (5 μm × 15 cm, 3 μm, 100 Å, Thermo Fisher Scientific, Waltham, MA, USA). Gradient elution was conducted at a flow rate of 4 μL/min using 80% acetonitrile as elution A and 0.1% FA as elution B. The procedure of gradient elution was set as follows: buffer B from 3 to 55% for 172 min, and buffer B from 55 to 98% for 5 min. An LTQ Orbitrap XL mass spectrometer (Thermo Fisher Scientific, San Jose, CA, USA) was used to analyze the MS and MS/MS of peptides. Each sample was measured for one time.

MS data were analyzed in MaxQuant software (Max Planck Institute of Biochemistry) by searching against the Uniprot database, and the parameters were set as follows: main search, 4.5 ppm; first search, 20 ppm; missed cleavage, 2; fixed modification, carbamidomethylation of cysteine; enzyme, trypsin^43^. The cutoff of FDR for peptide and protein identification was set to 0.01. Label-free quantitation algorithms were applied in MaxQuant. Total peptide signals within each run were normalized before quantitation. Afterwards, the counts of peptides between samples were compared to obtain a matrix of protein ratios, calculated as the median of all ratios for peptides^44^. Protein abundance was calculated on the basis of the normalized spectral protein intensity (LFQ intensity). OmicsBean (www.omicsbean.cn) was used to analyze the selected proteome data, in which GO analysis and KEGG pathway analysis were conducted^44^.

### Statistical analysis

The effects of diet alteration on the body weight, blood indexes, gut microbiota, metabolites and proteins in fecal samples were evaluated by repeated measures ANOVA in SPSS 21 (IBM, Armonk, NY, USA), in which diet was set as the within-subject factor and BMI was set as the between-subject factor. Pairwise differences between the means were compared by a LS Means method. FDR corrections of p values were applied in the fecal microbiota, metabolite and proteomics analyses. The differences were considered significant when the *p* values were smaller than 0.05. Principal component analysis (PCA) was applied to evaluate the variations in fecal microbiota and metabolites among samples using SIMCA software (version 14.1, Umetrics Software Inc., Sweden). Pearson’s and Spearman correlation analyses between the relative abundance of fecal microbiota and metabolites were performed using the SAS software (version 9.2, SAS Institute Inc., NC, USA). The results of ANOVA and correlation analysis were shown in Supplementary Table 10.

## Supplementary information

Supplementary files

## Data Availability

Raw data for fecal microbiota, metabolites and proteomics are available in the BioStudies (https://www.ebi.ac.uk/biostudies/) repository under the accession number S-BSST650.

## Code availability

Computer code used in this study is available upon request from the corresponding author.
