# Supplementary material for: Body weight index indicates the responses of the fecal microbiota, metabolome and proteome to beef/chicken-based diet alterations in Chinese volunteers
Source: NPJ Biofilms Microbiomes. 2022 Jul 12;8:56. doi: 10.1038/s41522-022-00319-7 (PMC9276758; doi:10.1038/s41522-022-00319-7)
Supplement: Supplementary file 1 — Supplementary files [file 41522_2022_319_MOESM1_ESM.pdf]

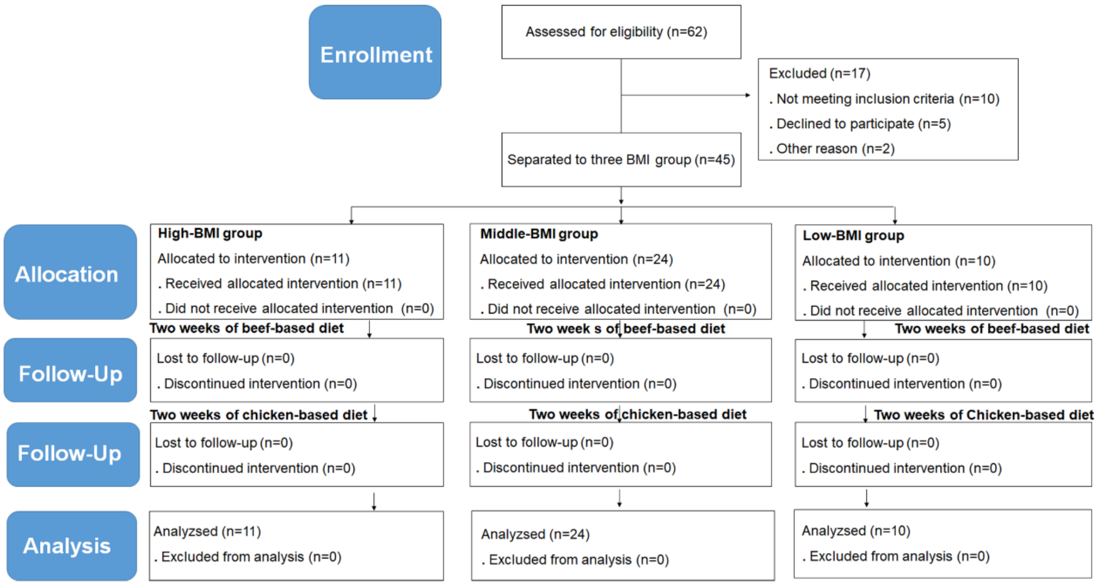

Supplementary Figure 1. CONSORT diagram depicting recruitment and selection of volunteers. Totally, 62 Chinese male candidates at Nanjing Agricultural University, aged from 18 to 25, responded to the recruitment. They signed the informed consent of the experimental project, conducted a daily habit questionnaire, and accepted physical examination. Finally, 45candidates were selected who had normal physical indicators, without access to antibiotics within three months and without serious medical history. They were assigned to high-BMI (n=11), middle-BMI (n=24 volunteers) and low-BMI (n=10 volunteers) groups which were provided with a beef-based diet for two weeks and subsequently with a chicken-based diet for another two weeks.

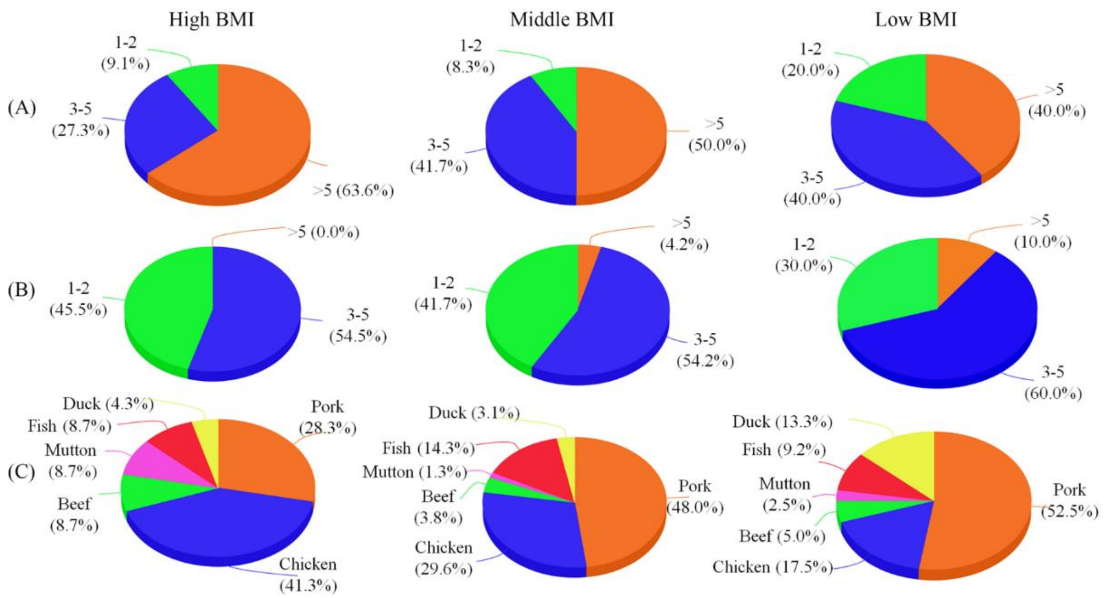

Supplementary Figure 2.Meat consumption and physical exercise habits of selected high-, middle- and low-BMI volunteers. (A) the frequency of meat consumption per week; (B) the frequency of physical exercise per week; (C) the choice of meat.

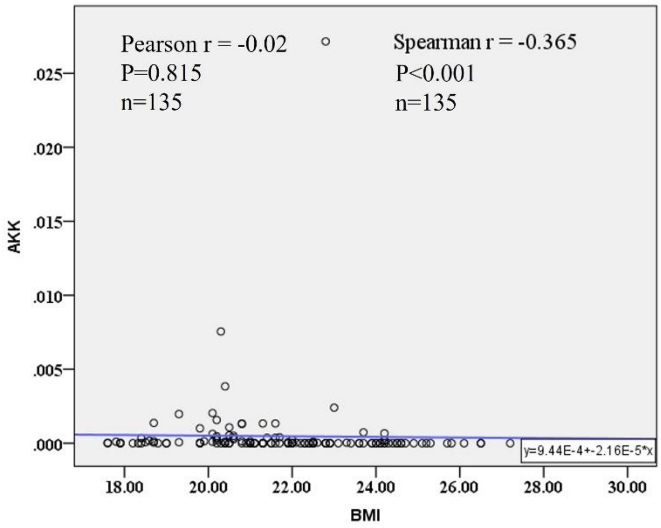

Supplementary Figure 3.Correlation between fecal Akkermansia abundance and host BMI using Pearson or Spearman correlation analyses.

Supplementary Table 1.Beef-based recipe for volunteers during the first 2 weeks of organized food ingestion.

Time	Breakfast	Lunch	Supper
Monday	Steamed bread (170 g)<br>pancakes (100 g)<br>eggs (60 g)<br>millet congee (260 g)	Stir-fried beef (113 g) with mushrooms (150 g)<br>stir-fried potato chips (200 g)<br>rice (379 g)<br>banana (150 g)	Stir-fried beef (97 g) with onion (150 g)<br>stir-fried tomato (200 g)<br>rice (243 g)<br>apple (200 g)
Tuesday	The same with Monday	Stir-fried beef (96 g) with cauliflower (150 g)<br>stir-fried spinach (200 g)<br>rice (385 g)<br>banana (150 g)	Stir-fried beef (83 g) with Chinese cabbage (150 g)<br>stir-fried lotus root slices (200 g)<br>rice (225 g)<br>orange (150 g)
Wednesday	The same with Monday	Stir-fried beef (101 g) with edible tree fungus (150 g)<br>stir-fried spinach (200 g)<br>rice (376 g)<br>banana (150 g)	Stir-fried beef (95 g) with green pepper (150 g)<br>stir-fried cucumber (200 g)<br>rice (272 g)<br>apple (200 g)
Thursday	The same with Monday	The same with Monday	The same with Monday
Friday	The same with Monday	The same with Wednesday	The same with Wednesday
Saturday	The same with Monday	The same with Tuesday	The same with Tuesday
Sunday	The same with Monday	The same with Wednesday	The same with Wednesday

Supplementary Table2.Chicken-based recipe for volunteers during the second 2 weeks of organized food ingestion.

Time	Breakfast	Lunch	Supper
Monday	Steamed bread (170 g)<br>pancakes (100 g)<br>eggs (60 g)<br>millet congee (260 g)	Stir-fried chicken (122 g) with mushrooms (150 g)<br>stir-fried potato chips (200 g)<br>rice (379 g)<br>banana (150 g)	Stir-fried chicken (104 g) with onion (150 g)<br>stir-fried tomato (200 g)<br>rice (243 g)<br>apple (200 g)
Tuesday	The same with Monday	Stir-fried chicken (103 g) with cauliflower (150 g)<br>stir-fried spinach (200 g)<br>rice (385 g)<br>banana (150 g)	Stir-fried chicken (89 g) with Chinese cabbage (150 g)<br>stir-fried lotus root slices (200 g)<br>rice (225 g)<br>orange (150 g)
Wednesday	The same with Monday	Stir-fried chicken (109 g) with edible tree fungus (150 g)<br>stir-fried spinach (200 g)<br>rice (376 g)<br>banana (150 g)	Stir-fried chicken (102 g) with green pepper (150 g)<br>stir-fried cucumber (200 g)<br>rice (272 g)<br>apple (200 g)
Thursday	The same with Monday	The same with Monday	The same with Monday
Friday	The same with Monday	The same with Wednesday	The same with Wednesday
Saturday	The same with Monday	The same with Tuesday	The same with Tuesday
Sunday	The same with Monday	The same with Wednesday	The same with Wednesday

Supplementary Table 3.Volunteers' information.

Volunteers' ID	Age	BMI	Frequency of meat consumption (per week)	Frequency of physical Exercise (per week)	Type of ingested meat
			High–BMI volunteers		
1	25	27.2	>5	3-5	Pork and chicken
2	23	25.7	>5	3-5	Beef and mutton
3	21	25.1	>5	1-2	Pork, beef, chicken and fish
4	27	24.5	>5	1-2	Pork and chicken
4	27	24.4	1-2	3-5	Chicken and fish
6	26	24.3	3-5	3-5	Pork and chicken
7	18	24.2	>5	3-5	Pork, beef, chicken and duck
8	19	24.2	3-5	1-2	Pork, beef, chicken and duck
9	24	24.1	>5	1-2	Chicken
10	26	24.1	>5	1-2	Pork and chicken
11	23	24.0	3-5	3-5	Pork and chicken
			Middle-BMI volunteers		
12	24	23.9	3-5	3-5	Chicken
13	22	22.5	>5	3-5	Pork, chicken and fish
14	22	22.5	3-5	3-5	Pork
15	25	22.5	3-5	3-5	Fish
16	25	22.5	3-5	1-2	Chicken
17	23	22.3	>5	3-5	Pork and fish
18	23	22.2	>5	3-5	Pork and chicken
19	23	22.0	>5	1-2	Pork and fish
20	26	22.0	>5	1-2	Pork, chicken and fish
21	20	21.7	3-5	1-2	Pork and chicken
22	22	21.7	>5	3-5	Duck and fish

23	23	21.3	1-2	1-2	Pork
24	23	21.3	>5	3-5	Pork and chicken
25	23	21.1	3-5	3-5	Pork, chicken and beef
26	22	21.1	>5	>5	Beef mutton and fish
27	23	20.9	>5	1-2	Pork and chicken
28	23	20.8	3-5	3-5	Chicken
29	22	20.8	>5	1-2	Pork and chicken
30	24	20.6	3-5	1-2	Pork, beef, chicken, and duck
31	23	20.6	3-5	1-2	Pork
32	21	20.5	>5	3-5	Pork and chicken
33	24	20.3	1-2	3-5	Pork
34	21	20.3	>5	1-2	Pork
35	22	20.1	3-5	3-5	Pork
Low BMI volunteers					
36	23	19.8	>5	1-2	Pork and chicken and duck
37	19	19.8	1-2	3-5	Beef, mutton, duck and fish
38	24	19.3	3-5	3-5	Pork
39	20	19.3	3-5	3-5	Pork and chicken
40	24	18.7	>5	>5	Pork, chicken and fish
41	23	18.7	>5	1-2	Pork, chicken and fish
42	23	18.5	3-5	1-2	Pork, chicken, beef and duck
43	24	18.2	1-2	3-5	Pork
44	21	17.9	3-5	3-5	Pork
45	23	17.6	>5	3-5	Pork and duck

Supplementary Table 4. Diversity indexes of fecal microbiota.

Groups	Diversity indexes		
	Chao	Shannon	Simpson
	Meat diet assays		
H1	788.60±135.06	3.55±0.51	0.09±0.06
H2	827.63±184.40	3.64±0.50	0.08±0.03
H3	823.00±220.35	3.39±0.87	0.07±0.02
M1	811.05±161.23	3.49±0.42	0.09±0.05
M2	864.14±186.09	3.65±0.47	0.07±0.04
M3	846.70±260.10	3.47±0.93	0.07±0.04
L1	836.65±155.30	3.55±0.34	0.08±0.04
L2	826.96±197.00	3.56±0.46	0.08±0.05
L3	834.64±148.42	3.56±0.27	0.08±0.02
	In vitro fecal microbiota incubation		
H1	539.45±121.97	3.44±0.57	0.10±0.06
H2	518.70±92.66	3.27±0.44	0.09±0.04
H3	563.70±108.42	3.37±0.39	0.09±0.04
M1	540.33±107.56	3.52±0.50	0.09±0.05
M2	523.33±97.89	3.20±0.43	0.10±0.05
M3	522.88±104.82	3.23±0.36	0.10±0.04
L1	536.44±61.18	3.53±0.28	0.08±0.03
L2	516.56±95.86	3.13±0.54	0.11±0.04
L3	483.00±67.30	2.99±0.42	0.13±0.06

H1, M1 and L1 refer to high-, middle- and low-BMI groups before meat-based diet alterations, H2, M2 and L2 refer to each group after beef-based diet and H3, M3 and L3 refer to each group after chicken-based diet.

Supplementary Table 5. Comparison of relative abundance of *Akkermansia muciniphila* during meat-based diet alteration.

Groups	Relative abundance of *Akkermansia* (%)		
	Control	Beef	Chicken
High-BMI	0.002±0.006	0.006±0.019	0.002±0.005
Middle-BMI	0.043±0.154	0.026±0.055	0.015±0.054
Low-BMI	0.051±0.075	0.040±0.050	0.036±0.067

Supplementary Table 6 Relative abundances (%) of core bacteria in feces of 45 volunteers after beef- and chicken-based diet alteration.

Items	Control	Beef	Chicken
Alistipes	1.65±1.89	1.41±1.60	1.36±1.45
Alloprevotella	1.56±3.66	1.17±2.73	1.47±3.44
Bacteroides	34.49±19.97	41.48±19.78	42.01±20.37
Faecalibacterium	6.31±5.09	5.95±4.18	5.36±4.32
Fusobacterium	1.10±3.88	1.06±2.31	1.54±3.53
Lachnoclostridium	2.16±3.14	1.68±1.90	1.56±2.17
Lachnospira	1.49±1.89[a]	2.67±3.01[b]	2.55±2.62[b]
Lachnospiraceae NK4A136 group	0.82±1.03[a]	1.65±2.00[b]	1.22±1.48[ab]
Parabacteroides	2.26±1.91	2.20±1.48	1.99±1.26
Phascolarctobacterium	1.46±2.08	1.76±1.85	1.57±1.60
Prevotella 9	12.45±21.44	9.94±19.12	9.29±18.48
Roseburia	2.56±1.88	2.46±1.50	2.71±1.96
Ruminococcus 2	1.01±1.14[a]	2.01±2.52[b]	1.95±2.62[b]
[Eubacterium] eligens group	1.08±1.65	1.32±1.36	1.37±1.49
[Eubacterium] rectale group	1.60±2.27	1.15±1.39	1.52±1.54

a, b, different superscripts denote significant differences among groups (P<0.05).

Supplementary Table 7. Relative abundance of the selected genera after incubation of fecal microbiota with beef or chicken protein digests for 24 h.

Genera	H1	H2	H3	M1	M2	M3	L1	L2	L3
Faecalibacterium	6.6 ±4.3$^{a, B}$	3.4 ±2.9^{b}	3.9 ±2.9^{b}	5.1 ±3.0$^{a, B}$	2.0 ±1.8^{b}	2.1 ±1.8^{b}	9.7 ±9.2$^{a, A}$	2.8 ±2.0^{b}	2.9 ±2.4^{b}
Megamonas	0.7 ±1.1	4.7 ±6.1^{AB}	7.0 ±9.7	0.7 ±3.2	1.6 ±3.6^{B}	2.2 ±4.7	0.1 ±0.3^{b}	8.5 ±17.3$^{a, A}$	8.2 ±16.1^{a}
Lachnoclostridium	1.5 ±1.0	0.9 ±0.7	0.8 ±0.6	2.3 ±3.4^{a}	0.8 ±0.5^{b}	0.8 ±0.5^{b}	3.0 ±4.6^{a}	0.6 ±0.5^{b}	0.5 ±0.4^{b}
Roseburia	2.6 ±1.8^{a}	0.3 ±0.3^{b}	0.3 ±0.3^{b}	3.0 ±2.3^{a}	0.3 ±0.3^{b}	0.3 ±0.3^{b}	2.7 ±0.8^{a}	0.4 ±0.5^{b}	0.2 ±0.3^{b}
Streptococcus	0.3 ±0.4^{b}	2.5 ±6.0^{ab}	3.3 ±6.9$^{a, A}$	0.7 ±2.5	0.6 ±1.2	0.7 ±1.6^{B}	0.7 ±1.0	0.9 ±1.9	1.0 ±1.8^{AB}
Ruminococcus_torques_group	0.3 ±0.2	1.0 ±0.7	1.3 ±1.9^{AB}	0.4 ±0.3	0.7 ±1.7	1.0 ±2.3^{B}	0.6 ±0.7	1.5 ±1.6	2.7 ±3.6^{A}
Collinsella	0.1 ±0.1^{b}	1.5 ±3.1^{a}	0.7 ±1.0^{ab}	0.2 ±0.2^{b}	1.1 ±2.0^{a}	1.1 ±1.3^{a}	0.1 ±0.1^{b}	1.4 ±1.0^{a}	0.2 ±0.1^{b}
Lachnospira	1.0 ±0.7^{B}	0.4 ±0.4	0.4 ±0.5	1.9 ±2.4$^{a, A}$	0.3 ±0.4^{b}	0.3 ±0.3^{b}	1.1 ±1.4^{AB}	0.3 ±0.4	0.3 ±0.4
Eubacterium rectale group	0.8 ±0.6^{B}	0.1 ±0.2	0.2 ±0.3	1.7 ±2.6$^{a, A}$	0.3 ±0.5^{b}	0.3 ±0.5^{b}	1.2 ±0.8^{AB}	0.5 ±0.9	0.6 ±1.0
Lachnospiraceae uncultured	0.7 ±0.5^{B}	0.4 ±0.3	0.4 ±0.2	1.1 ±0.9$^{a, AB}$	0.4 ±0.7^{b}	0.4 ±0.7^{b}	1.6 ±2.2$^{a, A}$	0.3 ±0.3^{b}	0.3 ±0.4^{b}
Eubacterium eligens group	1.3 ±1.5^{a}	0.4 ±0.7$^{b, A}$	0.4 ±0.6^{ab}	1.0 ±1.9^{a}	0.1 ±0.1$^{b, B}$	0.1 ±0.1^{b}	0.9 ±1.5^{a}	0.2 ±0.2$^{b, AB}$	0.1 ±0.1^{a}
Bacteroidales_S24_7_group_norank	0.3 ±0.9^{B}	0.2 ±0.6	0.3 ±1.0	0.3 ±0.5^{B}	0.1 ±0.2	0.1 ±0.3	2.3 ±2.4$^{a, A}$	0.8 ±1.0^{b}	1.1 ±1.5^{ab}
Subdoligranulum	1.3 ±2.0$^{a, A}$	0.4 ±0.7^{b}	0.4 ±0.6^{b}	0.4 ±0.4^{B}	0.2 ±0.3	0.3 ±0.5	0.5 ±0.8^{B}	0.3 ±0.3	0.4 ±0.8
Dorea	0.2 ±0.1	0.5 ±0.7	0.3 ±0.2^{B}	0.3 ±0.2	0.4 ±0.5	0.2 ±0.3^{B}	0.3 ±0.2^{b}	0.5 ±0.7^{ab}	0.8 ±0.9$^{a, A}$
Lachnospiraceae_NK4A136_group	0.4 ±0.6^{B}	0.1 ±0.1	0.1 ±0.1	0.9 ±1.1$^{a, B}$	0.1 ±0.1^{b}	0.1 ±0.1^{b}	1.1 ±1.4$^{a, A}$	0.1 ±0.2^{b}	0.1 ±0.2^{b}

Fusicatenibacter	0.5 ± 1.1	0.2 ± 0.2	0.1 ± 0.2	0.6 ± 0.7^{a}	0.1 ± 0.2^{b}	0.2 ± 0.2^{b}	0.6 ± 0.6	0.2 ± 0.2	0.2 ± 0.2
Eubacterium__coprostanoligenes	0.3 ± 0.4	0.1 ± 0.2	0.1 ± 0.2	0.6 ± 0.8^{a}	0.1 ± 0.2^{b}	0.1 ± 0.2^{b}	0.6 ± 1.0^{a}	0.1 ± 0.1^{b}	0.1 ± 0.1^{b}
Ruminococcus_1	0.4 ± 0.5	0.2 ± 0.4^{AB}	0.2 ± 0.3	0.5 ± 0.6^{a}	$0.1\pm0.1^{b, B}$	0.1 ± 0.1^{b}	0.4 ± 0.3	0.4 ± 0.9^{A}	0.3 ± 0.5
Erysipelotrichaceae_UCG_003	0.2 ± 0.2^{b}	$0.7\pm1.1^{a, A}$	0.3 ± 0.4^{ab}	0.1 ± 0.1	0.2 ± 0.3^{B}	0.2 ± 0.4	0.3 ± 0.4	0.3 ± 0.4^{AB}	0.3 ± 0.4
Lachnospiraceae_UCG_004	0.3 ± 0.2^{B}	0.2 ± 0.1	0.1 ± 0.1	$0.5\pm0.4^{a, AB}$	0.2 ± 0.1^{b}	0.2 ± 0.2^{b}	$0.8\pm0.8^{a, A}$	0.1 ± 0.1^{b}	0.1 ± 0.1^{b}
Haemophilus	$0.5\pm0.7^{a,}$	0.1 ± 0.1^{b}	0.1 ± 0.1^{b}	$0.5\pm0.8^{a,}$	0.1 ± 0.4^{b}	0.1 ± 0.4^{b}	0.4 ± 0.3^{a}	0.03 ± 0.03^{b}	0.03 ± 0.03^{b}

H1, M1 and L1 refer to high-, middle- and low-BMI groups before meat-based diet alterations, H2, M2 and L2 refer to each group after beef-based diet and H3, M3 and L3 refer to each group after chicken-based diet. Different superscript lowercases (a and b) denote significant differences induced by meat-based diet alteration in different BMI volunteers ($P<0.05$). Different superscript uppercases (A and B) denote significant differences under the same diet among different BMI volunteers ($P<0.05$).

Supplementary Table 8. Number of average and total OTUs in each groups illustrated in Figure 2A and 2B.

Samples	Number of average OTUs	Number of OTUs										
		Bacteroidota	Firmicutes	Proteobacteria	Actinobacteria	Cyanobacteria	Fusobacteria	Verrucomicrobiota	Desulfobacterota	Elusimicrobiota	Synergistota	Others
Beef (in vivo) vs Chicken (in vivo)												
Unique for beef group	215 ±58	240	169	4	3	1	1	—	—	—	—	1
Unique for chicken group	185 ±57	197	172	6	2	—	—	—	—	—	—	—
Shared	381 ±87	1403	1279	57	48	5	6	6	11	1	5	12
Beef (in vitro) vs Chicken (in vitro)												
Unique for beef group	175 ±59	333	198	11	5	—	—	1	1	—	—	—
Unique for chicken group	128 ±38	179	182	1	3	1	—	—	—	—	—	1
Shared	344 ±73	1278	889	57	46	4	6	5	10	—	4	9
Beef (in vivo) vs Beef (in vitro)												
Unique for in vivo	286 ±86	306	518	8	6	2	1	1	—	1	1	—
Unique for in vitro	208 ±95	268	157	14	7	—	—	1	—	—	—	1
Shared	314 ±82	1335	932	53	45	4	6	5	11	—	4	14
Chicken (in vivo) vs Chicken (in vitro)												

Unique for in vivo	268 ± 80	219	642	12	5	2	—	1	1	1	—	3
Unique for in vitro	183 ± 73	183	135	7	3	2	—	1	—	—	—	—
Shared	292 ± 88	1384	807	51	48	3	6	4	10	0	4	27

Supplementary Table 9. Fecal metabolites that were identified to be significantly different after beef- and chicken-based diets.

Compounds	Intensity-beef	Intensity-chicken
11H-14,15-EETA	2337.85±2647.69	1288.68±1488.80
Proline	32689.11±13152.16	24411.47±14083.62
L-Methionine	1143.24±986.07	1880.73±1301.49
L-Aminoadipate	1839.70±887.52	2467.33±1333.10
Hexadecanoic acid	7689.92±4908.71	4670.98±1934.90
Prostaglandin G2	1172.49±334.63	1565.29±523.02
Hypoxanthine	3302.81±2248.66	5625.98±5393.71
Deoxyguanosine	3417.05±645.13	2939.03±827.36
Deoxyuridine	8569.69±7989.06	4887.35±3158.74
Cytidine	6694.30±2925.52	4864.85±2447.16
Thymine	2920.44±2852.84	1480.09±1473.68
Methoxy-hydroxyphenylglycolaldehyde	903.68±424.02	1437.76±1112.26
Dihydroxyphenylacetaldehyde	2182.24±1243.18	3837.50±2593.16
Methoxy-hydroxyphenylacetaldehyde	9070.27±9573.86	16195.68±16921.68
FMNH	7064.28±3093.19	3107.09±1298.66
FMNH2	4855.51±1067.48	3997.80±1261.57
Nicotinic acid	9376.89±5193.08	6663.13±3785.69

Supplementary Table 10. Data analysis tables of body weight, blood indexes, fecal microbiota, metabolites and proteomics.

Supplementary Table 10-1. P values of body weight and blood indexes.

Items	Diet	BMI	Diet*BMI
Body weight (kg)	0.13	0.15	0.87
Systolic blood pressure (mm Hg)	0.25	0.55	0.54
Diastolic blood pressure (mm Hg)	0.33	0.68	0.60
Total cholesterol (mM)	<0.001	0.20	0.66
Triglyceride (mM)	0.02	0.19	0.09
High density lipoprotein (mM)	0.28	0.29	0.52
Low density lipoprotein (mM)	<0.001	0.27	0.39
Blood glucose (mM)	0.07	0.14	0.27
Leucocytes (10^9/L)	0.70	0.09	0.90
Lymphocytes (%)	0.63	0.72	0.96
Monocytes (%)	<0.001	0.38	0.76
Neutrophils (%)	0.26	0.67	0.85
Eosinophils (%)	0.54	0.94	0.62
Basophils (%)	<0.001	0.76	0.08
Giant immature cell (%)	0.06	0.69	0.33
Erythrocytes (10^{12}/L)	0.02	0.46	0.55
Hemoglobin (g/L)	0.10	0.63	0.22
Hematocrit	<0.001	0.81	0.28
Mean corpuscular volume (fL)	0.06	0.29	0.26
Mean corpuscular hemoglobin (pg)	0.18	0.09	0.87
Mean corpuscular hemoglobin concentration (g/L)	<0.001	0.44	0.70
Red blood cell distribution width (%)	0.06	0.09	0.35
Platelet (10^9/L)	0.09	0.29	0.80
Mean platelet volume (fL)	<0.001	0.25	0.57
Platelet distribution width (%)	<0.001	0.27	0.68

Supplementary Table 10-2. P values of two-way repeated measures ANOVA of fecal microbiota at the phylum level

Microbiota	Diet	BMI	diet*BMI
Actinobacteria	0.19	0.57	0.44
Bacteroidetes	0.11	0.29	0.58
Cyanobacteria	0.42	0.36	0.17
Cyanobacteria	0.27	0.94	0.39
Elusimicrobia	0.72	0.66	0.79
Epsilonbacteraeot	0.16	0.48	0.03
Firmicutes	0.17	0.57	0.44
Fusobacteria	0.82	0.17	0.90
Lentisphaerae	0.12	0.45	0.29
Patescibacteria	0.54	0.22	0.34
Proteobacteria	0.06	0.34	0.80
Synergistetes	0.30	0.34	0.23
Tenericutes	0.89	0.70	0.79
Unclassified	0.52	0.63	0.62
Verrucomicrobia	0.75	0.44	0.80
F/B	0.92	0.88	0.64

Supplementary Table 10-3. P values of two-way repeated measures ANOVA of fecal microbiota at the genus level before FDR correction.

Microbiota	diet	BMI	diet*BMI
Bacteroides	0.22	0.32	0.45
Prevotella 9	0.74	0.33	0.75
Faecalibacterium	0.11	0.66	0.02
Roseburia	0.79	0.52	0.98
Lachnospira	0.004	0.71	0.14
Parabacteroides	0.71	0.76	0.86
Lachnoclostridium	0.46	0.84	0.70
Ruminococcus 2	0.32	0.32	0.19
Phascolarctobacterium	0.31	0.62	0.26
Prevotella 2	0.61	0.49	0.99
Agathobacter	0.47	0.47	0.88
Alistipes	0.37	0.90	0.87
Alloprevotella	0.86	0.25	0.93
Lachnospiraceae NK4A136 group	0.03	0.33	0.93
Fusobacterium	0.83	0.06	0.97
Blautia	0.006	0.57	0.75
Dialister	0.01	0.96	0.62
Parasutterella	0.22	0.10	0.56
Muribaculaceae_norank	0.27	0.04	0.61
Ruminococcaceae UCG-002	0.63	0.66	0.43
Lachnospiraceae_uncultured	0.13	0.70	0.38
Megamonas	0.30	0.08	0.17
Escherichia-Shigella	0.22	0.31	0.79
Sutterella	0.63	0.38	0.99
Megasphaera	0.27	0.26	0.72
Bifidobacterium	0.44	0.47	0.76

Subdoligranulum	0.31	0.30	0.27
Paraprevotella	0.67	0.10	0.44
Ruminococcus 1	0.80	0.56	0.88
Haemophilus	0.07	0.59	0.77
Fusicatenibacter	0.01	0.46	0.96
Lachnospiraceae UCG-004	0.02	0.07	0.24
Ruminococcaceae UCG-014	0.61	0.09	0.74
Streptococcus	0.20	0.68	0.96
Anaerostipes	0.23	0.74	0.92
Coprococcus 2	0.84	0.34	0.96
Romboutsia	0.40	0.57	0.27
Ruminococcaceae UCG-005	0.04	0.10	0.39
Ruminococcaceae UCG-003	0.42	0.55	0.33
Lachnospiraceae UCG-001	0.21	0.14	0.51
Butyricicoccus	0.04	0.75	0.87
Bilophila	0.10	0.71	0.75
Ruminococcaceae NK4A214 group	0.55	0.91	0.25
Odoribacter	0.81	0.27	0.96
Dorea	0.30	0.45	0.48
Butyricimonas	0.23	0.52	0.68
Christensenellaceae R-7 group	0.90	0.20	0.59
Coprobacter	0.02	0.30	0.31
Erysipelotrichaceae UCG-003	0.23	0.04	0.25
Klebsiella	0.02	0.70	0.52
Barnesiella	0.99	0.33	0.96
Enterococcus	0.77	0.51	0.88
Mitsuokella	0.42	0.43	0.70
Ruminococcaceae UCG-013	0.02	0.55	0.82
Mollicutes RF39_norank	0.88	0.70	0.87

Veillonella	0.35	0.99	0.32
Prevotellaceae_uncultured	0.20	0.24	0.18
Clostridium sensu stricto 1	0.48	0.88	0.45
Lachnospiraceae ND3007 group	0.76	0.55	0.91
Ruminococcaceae_uncultured	0.96	0.91	0.71
Tyzzerella 3	0.28	0.04	0.61
Lachnospiraceae UCG-003	0.52	0.52	0.47
Flavonifractor	0.52	0.88	0.57
Coprococcus 3	0.24	0.54	0.99
Succinivibrio	0.20	0.22	0.19
Collinsella	0.22	0.41	0.20
Lactobacillus	0.22	0.58	0.84
Ruminiclostridium 6	0.11	0.80	0.73
Ruminiclostridium 9	0.12	0.79	0.32
CAG-56	0.56	0.30	0.99
Rhodospirillales_norank	0.64	0.09	0.84
Intestinibacter	0.25	0.85	0.43
Holdemanella	0.23	0.88	0.69
Oscillibacter	0.86	0.72	0.15
Ruminiclostridium 5	0.77	0.57	0.86
Butyrivibrio	0.31	0.26	0.15
Prevotella 7	0.87	0.45	0.96
Desulfovibrio	0.71	0.47	0.99
Ruminococcaceae UCG-010	0.39	0.92	0.71
Akkermansia	0.75	0.42	0.81
Gastranaerophilales_norank	0.50	0.29	0.14
Lachnospiraceae UCG-010	0.007	0.16	0.08
Coprococcus 1	0.28	0.68	0.47
Allisonella	0.08	0.26	0.12

Prevotellaceae NK3B31 group	0.73	0.77	0.80
Lachnospiraceae_Unclassified	0.08	0.12	0.54
Hungatella	0.77	0.38	0.93
Acidaminococcus	0.74	0.41	0.87
Tyzzerella 4	0.07	0.79	0.83
Bacteroidales_norank	0.86	0.67	0.17
Catenibacterium	0.02	0.17	0.46
Elusimicrobium	0.74	0.61	0.82
Terrisporobacter	0.26	0.48	0.12
Oxalobacter	0.0003	0.63	0.10
Turicibacter	0.02	0.07	0.04
UBA1819	0.06	0.22	0.43
Lachnospiraceae FCS020 group	0.04	0.48	0.83
Lachnospiraceae UCG-008	0.84	0.60	0.90
Faecalitalea	0.17	0.21	0.14
Prevotellaceae UCG-001	0.46	0.31	0.15
Adlercreutzia	0.33	0.48	0.94
Family XIII AD3011 group	0.24	0.76	0.54
Erysipelatoclostridium	0.74	0.14	0.93
GCA-900066575	0.23	0.83	0.68
Marvinbryantia	0.78	0.30	0.28
Peptococcus	0.71	0.51	0.96
Holdemania	0.66	0.43	0.33
Unclassified	0.55	0.61	0.66
Family XIII UCG-001	0.56	0.57	0.49
Clostridiales vadinBB60 group_norank	0.19	0.34	0.63
CAG-352	0.64	0.37	0.88
Rikenellaceae RC9 gut group	0.24	0.22	0.20
Eggerthella	0.60	0.34	0.82

Tyzzerella	0.21	0.82	0.57
Prevotellaceae UCG-004	0.29	0.22	0.15
Ruminococcaceae UCG-004	0.30	0.44	0.10
Negativibacillus	0.18	0.66	0.60
Selenomonas	0.76	0.63	0.85
Phocea	0.02	0.34	0.41
Fournierella	0.73	0.69	0.94
Aggregatibacter	0.03	0.73	0.62
Actinomyces	0.26	0.67	0.47
Intestinimonas	0.23	0.42	0.14
Ruminococcaceae UCG-009	0.26	0.38	0.60
Flavobacteriaceae_uncultured	0.79	0.31	1.00
Lachnospiraceae_norank	0.04	0.19	0.08
Hafnia-Obesumbacterium	0.07	0.11	0.02
Comamonas	0.27	0.41	0.25
Halomonas	0.004	0.51	0.007
Brevundimonas	0.25	0.17	0.22
Pyramidobacter	0.26	0.23	0.21
Gemella	0.43	0.56	0.69
Cellulosilyticum	0.19	0.53	0.75
Chloroplast_norank	0.72	0.68	0.76
Oscillospira	0.26	0.25	0.18
Mailhella	0.53	0.16	0.65
Porphyromonas	0.06	0.57	0.46
Peptococcaceae_uncultured	0.08	0.02	0.03
Christensenellaceae_uncultured	0.76	0.08	0.92
Anaerotruncus	0.81	0.19	0.96
Candidatus Soleaferrea	0.88	0.46	0.24
Desulfovibrionaceae_uncultured	0.05	0.44	0.83

Leifsonia	0.51	0.64	0.70
Saccharimonadaceae_norank	0.54	0.26	0.39
Olsenella	0.28	0.82	0.55
Rhodococcus	0.79	0.61	0.98
Cloacibacillus	0.86	0.20	0.96
Ezakiella	0.10	0.30	0.22
Senegalimassilia	0.60	0.41	0.93
Prevotella	0.18	0.43	0.47
Prevotella 6	0.66	0.24	0.50
Moryella	0.19	0.66	0.15
Lactococcus	0.03	0.28	0.22
Eisenbergiella	0.91	0.26	0.97
Coriobacteriales Incertae Sedis_uncultured	0.04	0.63	0.75
Oribacterium	0.49	0.25	0.37
Ruminiclostridium	0.77	0.04	0.87
Howardella	0.90	0.43	0.85
Dielma	0.82	0.28	0.97
Pseudomonas	0.62	0.52	0.36
Enterorhabdus	0.12	0.16	0.12
Sellimonas	0.92	0.73	0.61
Ruminococcaceae UCG-008	0.23	0.17	0.34
Epulopiscium	0.52	0.74	0.20
Erysipelotrichaceae UCG-004	0.57	0.15	0.29
Defluviitaleaceae UCG-011	0.86	0.33	0.87
Eubacterium	0.81	0.57	0.93
Campylobacter	0.17	0.48	0.03
Rothia	0.72	0.58	0.80
Hydrogenoanaerobacterium	0.84	0.58	0.49
Shuttleworthia	0.61	0.50	0.64

Peptostreptococcus	0.27	0.98	0.83
Neisseria	0.10	0.41	0.43
Izimaplasmatales_norank	0.10	0.22	0.18
Candidatus Stoquefichus	0.77	0.64	0.88
Slackia	0.46	0.56	0.81
Morganella	0.36	0.32	0.11
Parvimonas	0.18	0.38	0.11
Sanguibacteroides	0.31	0.57	0.80
Solobacterium	0.83	0.79	0.25
Catabacter	0.66	0.09	0.89
Aliihoeflea	0.006	0.45	0.29
Peptostreptococcaceae_uncultured	0.21	0.32	0.831
Ruminococcaceae_Unclassified	0.04	0.32	0.60
GCA-900066225	0.75	0.35	0.83
Microbacterium	0.95	0.52	0.83
UC5-1-2E3	0.76	0.34	0.73
Gordonibacter	0.35	0.90	0.87
Acetitomaculum	0.93	0.26	0.90
Pelagibacterium	0.86	0.23	0.70
Christensenella	0.74	0.65	0.34
GCA-900066755	0.22	0.25	0.80
Aeromonas	0.03	0.22	0.19
Lachnospiraceae NK3A20 group	0.20	0.22	0.19
DTU014_norank	0.81	0.48	0.88
Mucispirillum	0.001	0.005	<0.0001
Anaerofilum	0.24	0.15	0.67
Sharpea	0.81	0.45	0.93
Atopobiaceae_uncultured	0.19	0.08	0.04
Mogibacterium	0.86	0.12	0.73

Abiotrophia	0.71	0.79	0.60
Leuconostoc	0.002	0.20	0.17
DTU089	0.82	0.61	0.20
Paraclostridium	0.33	0.07	0.58
Mitochondria_norank	1.00	0.63	0.23
Harryflintia	0.69	0.44	0.93
Lachnoanaerobaculum	1.00	0.15	0.18
Vagococcus	0.62	0.47	0.69
Peptoniphilus	0.25	0.22	0.29
Parvibacter	0.38	0.40	0.30
Ruminococcaceae UCG-011	0.29	0.56	0.58
Raoultibacter	0.56	0.30	0.69
Staphylococcus	0.34	0.19	0.25
Faecalicoccus	0.36	0.78	0.89
Methylobacterium	1.00	0.84	0.65
Erysipelotrichaceae UCG-006	0.20	0.22	0.19
Acinetobacter	0.85	0.16	0.85
Papillibacter	1.00	0.76	0.63
Corynebacterium	0.57	0.53	0.82
Proteus	0.37	0.43	0.15
F0332	0.03	0.86	0.75
Lactonifactor	0.03	0.56	0.22
Selenomonas 3	0.38	0.01	0.16
Rikenella	0.66	0.32	0.92
Asteroleplasma	0.20	0.22	0.19
Atopobium	0.36	0.45	0.86
Cardiobacterium	1.00	0.14	1.00
Dubosiella	0.14	0.54	0.73
Acetanaerobacterium	0.57	0.36	0.72

Anaerococcus	0.23	0.70	0.11
Anaerofustis	0.10	0.51	0.70
Nesterenkonia	0.04	0.14	0.68
Ruminiclostridium 1	0.56	0.29	0.64
Saccharimonadales_norank	0.40	0.41	0.99
Chelativorans	0.07	0.51	0.23
Coriobacteriales_norank	0.19	0.62	0.74
Merdibacter	0.85	0.42	0.93
Tenacibaculum	0.21	0.42	0.39
Enorma	0.14	0.18	0.13
A2	0.10	0.78	0.29
Angelakisella	0.61	0.60	0.20
Corynebacterium 1	0.34	0.65	0.75
Johnsonella	0.14	0.65	0.78
Stomatobaculum	1.00	0.26	1.00
Anaeroplasma	0.20	0.24	0.19
Finegoldia	0.19	0.62	0.74
Leptotrichia	0.72	0.66	0.79
MBA03_norank	1.00	0.42	1.00
Murdochiella	0.19	0.62	0.74

Supplementary Table 10-4. P values of two-way repeated measures ANOVA of fecal microbiota at the genus level after FDR correction.

Microbiota	diet	BMI	Diet*BMI
Oxalobacter	0.08	0.83	1.00
Mucispirillum	0.13	1.00	0.03
Leuconostoc	0.17	1.00	1.00
Halomonas	0.25	0.86	0.88
Lachnospira	0.20	0.83	1.00
Aliihoeflea	0.25	0.87	1.00
Blautia	0.21	0.84	1.00
Lachnospiraceae UCG-010	0.22	1.00	1.00
Dialister	0.33	0.98	1.00
Fusicatenibacter	0.35	0.86	1.00
Klebsiella	0.34	0.84	1.00
Ruminococcaceae UCG-013	0.35	0.86	1.00
Phocea	0.39	0.87	1.00
Lachnospiraceae UCG-004	0.39	1.00	1.00
Catenibacterium	0.38	1.00	1.00
Coprobacter	0.36	0.92	1.00
Turicibacter	0.34	1.00	1.00
Lactonifactor	0.35	0.84	1.00
Lachnospiraceae NK4A136 group	0.36	0.88	1.00
Aeromonas	0.35	1.00	1.00
F0332	0.35	0.91	1.00
Aggregatibacter	0.34	0.84	1.00
Lactococcus	0.35	0.94	1.00
Ruminococcaceae_Unclassified	0.38	0.89	1.00
Ruminococcaceae UCG-005	0.38	1.00	1.00
Coriobacteriales Incertae	0.38	0.82	1.00

Sedis_uncultured			
Butyricicoccus	0.37	0.86	1.00
Nesterenkonia	0.38	1.00	1.00
Lachnospiraceae_norank	0.41	1.00	1.00
Lachnospiraceae FCS020 group	0.41	0.86	1.00
Desulfovibrionaceae_uncultured	0.44	0.87	1.00
Porphyromonas	0.44	0.83	1.00
UBA1819	0.46	1.00	1.00
Hafnia-Obesumbacterium	0.48	1.00	1.00
Tyzzerella 4	0.47	0.86	1.00
Chelativorans	0.47	0.85	1.00
Haemophilus	0.47	0.83	1.00
Lachnospiraceae_Unclassified	0.53	1.00	1.00
Allisonella	0.51	0.95	1.00
Peptococcaceae_uncultured	0.53	1.00	1.00
Bilophila	0.58	0.83	1.00
Ezakiella	0.57	0.95	1.00
Neisseria	0.56	0.91	1.00
A2	0.56	0.87	1.00
Izimaplasmatales_norank	0.56	1.00	1.00
Anaerofustis	0.57	0.86	1.00
Ruminiclostridium 6	0.60	0.87	1.00
Faecalibacterium	0.59	0.83	1.00
Enterorhabdus	0.63	1.00	1.00
Ruminiclostridium 9	0.62	0.87	1.00
Lachnospiraceae_uncultured	0.63	0.83	1.00
Dubosiella	0.65	0.85	1.00
Enorma	0.67	1.00	1.00
Johnsonella	0.66	0.82	1.00

Campylobacter	0.75	0.85	1.00
Faecalitalea	0.77	1.00	1.00
Parvimonas	0.77	0.91	1.00
Prevotella	0.77	0.87	1.00
Negativibacillus	0.76	0.83	1.00
Moryella	0.77	0.82	1.00
Atopobiaceae_uncultured	0.77	1.00	1.00
Clostridiales vadinBB60 group_norank	0.76	0.86	1.00
Coriobacteriales_norank	0.75	0.83	1.00
Finegoldia	0.74	0.83	1.00
Murdochiella	0.73	0.82	1.00
Cellulosilyticum	0.73	0.84	1.00
Streptococcus	0.74	0.82	1.00
Succinivibrio	0.73	1.00	1.00
Anaeroplasma	0.73	1.00	1.00
Asteroleplasma	0.71	1.00	1.00
Erysipelotrichaceae UCG-006	0.70	1.00	1.00
Lachnospiraceae NK3A20 group	0.69	1.00	1.00
Prevotellaceae_uncultured	0.70	1.00	1.00
Lachnospiraceae UCG-001	0.70	1.00	1.00
Peptostreptococcaceae_uncultured	0.69	0.91	1.00
Tyzzerella	0.69	0.88	1.00
Tenacibaculum	0.69	0.91	1.00
Bacteroides	0.69	0.88	1.00
GCA-900066755	0.69	0.99	1.00
Collinsella	0.69	0.93	1.00
Lactobacillus	0.68	0.82	1.00
Escherichia-Shigella	0.68	0.92	1.00
Parasutterella	0.67	1.00	1.00

Anaerococcus	0.68	0.83	1.00
GCA-900066575	0.67	0.89	1.00
Intestinimonas	0.66	0.89	1.00
Ruminococcaceae UCG-008	0.66	1.00	1.00
Holdemanella	0.66	0.92	1.00
Butyricimonas	0.65	0.85	1.00
Erysipelotrichaceae UCG-003	0.64	1.00	1.00
Anaerostipes	0.64	0.85	1.00
Anaerofilum	0.64	1.00	1.00
Family XIII AD3011 group	0.64	0.86	1.00
Rikenellaceae RC9 gut group	0.64	1.00	1.00
Coprococcus 3	0.64	0.85	1.00
Peptoniphilus	0.64	1.00	1.00
Brevundimonas	0.64	1.00	1.00
Intestinibacter	0.64	0.90	1.00
Actinomyces	0.64	0.83	1.00
Oscillospira	0.65	1.00	1.00
Pyramidobacter	0.64	1.00	1.00
Ruminococcaceae UCG-009	0.64	0.90	1.00
Terrisporobacter	0.64	0.85	1.00
Muribaculaceae_norank	0.64	1.00	1.00
Megasphaera	0.64	0.98	1.00
Comamonas	0.64	0.93	1.00
Peptostreptococcus	0.64	0.99	1.00
Tyzzerella 3	0.64	1.00	1.00
Coprococcus 1	0.64	0.83	1.00
Olsenella	0.65	0.88	1.00
Ruminococcaceae UCG-011	0.64	0.85	1.00
Prevotellaceae UCG-004	0.66	1.00	1.00

Dorea	0.66	0.87	1.00
Megamonas	0.66	1.00	1.00
Ruminococcaceae UCG-004	0.65	0.88	1.00
Butyrivibrio	0.66	0.96	1.00
Sanguibacteroides	0.67	0.84	1.00
Phascolarctobacterium	0.66	0.82	1.00
Subdoligranulum	0.66	0.96	1.00
Ruminococcus 2	0.68	0.89	1.00
Adlercreutzia	0.67	0.86	1.00
Paraclostridium	0.67	1.00	1.00
Corynebacterium 1	0.68	0.83	1.00
Staphylococcus	0.68	1.00	1.00
Veillonella	0.69	0.99	1.00
Gordonibacter	0.69	0.93	1.00
Faecalicoccus	0.70	0.87	1.00
Morganella	0.70	0.92	1.00
Atopobium	0.70	0.86	1.00
Proteus	0.71	0.89	1.00
Alistipes	0.70	0.93	1.00
Selenomonas 3	0.71	1.00	1.00
Parvibacter	0.72	0.92	1.00
Ruminococcaceae UCG-010	0.74	0.94	1.00
Saccharimonadales_norank	0.73	0.91	1.00
Romboutsia	0.73	0.83	1.00
Ruminococcaceae UCG-003	0.76	0.85	1.00
Mitsuokella	0.76	0.88	1.00
Gemella	0.77	0.85	1.00
Bifidobacterium	0.79	0.87	1.00
Slackia	0.81	0.84	1.00

Lachnoclostridium	0.81	0.89	1.00
Prevotellaceae UCG-001	0.80	0.92	1.00
Agathobacter	0.81	0.86	1.00
Clostridium sensu stricto 1	0.82	0.91	1.00
Oribacterium	0.83	1.00	1.00
Gastranaerophilales_norank	0.85	0.96	1.00
Leifsonia	0.87	0.83	1.00
Epulopiscium	0.87	0.85	1.00
Flavonifractor	0.87	0.91	1.00
Lachnospiraceae UCG-003	0.87	0.85	1.00
Mailhella	0.88	1.00	1.00
Saccharimonadaceae_norank	0.88	0.97	1.00
Unclassified	0.89	0.84	1.00
Ruminococcaceae NK4A214 group	0.88	0.93	1.00
Ruminiclostridium 1	0.89	0.95	1.00
Family XIII UCG-001	0.89	0.82	1.00
CAG-56	0.89	0.93	1.00
Raoultibacter	0.88	0.94	1.00
Erysipelotrichaceae UCG-004	0.89	1.00	1.00
Acetanaerobacterium	0.89	0.88	1.00
Corynebacterium	0.88	0.84	1.00
Eggerthella	0.92	0.87	1.00
Senegalimassilia	0.92	0.91	1.00
Ruminococcaceae UCG-014	0.92	1.00	1.00
Shuttleworthia	0.91	0.87	1.00
Prevotella 2	0.91	0.84	1.00
Angelakisella	0.91	0.84	1.00
Pseudomonas	0.91	0.85	1.00
Vagococcus	0.91	0.87	1.00

Ruminococcaceae UCG-002	0.92	0.82	1.00
Sutterella	0.92	0.90	1.00
CAG-352	0.92	0.90	1.00
Rhodospirillales_norank	0.92	1.00	1.00
Catabacter	0.94	1.00	1.00
Prevotella 6	0.94	1.00	1.00
Rikenella	0.94	0.90	1.00
Holdemania	0.93	0.89	1.00
Paraprevotella	0.93	1.00	1.00
Harryflintia	0.96	0.88	1.00
Parabacteroides	0.98	0.86	1.00
Desulfovibrio	0.97	0.86	1.00
Abiotrophia	0.97	0.87	1.00
Peptococcus	0.97	0.87	1.00
Chloroplast_norank	0.97	0.82	1.00
Leptotrichia	0.97	0.83	1.00
Rothia	0.97	0.82	1.00
Prevotellaceae NK3B31 group	0.96	0.86	1.00
Fournierella	0.96	0.83	1.00
Christensenella	0.97	0.82	1.00
Acidaminococcus	0.97	0.92	1.00
Prevotella 9	0.96	0.88	1.00
Erysipelatoclostridium	0.96	1.00	1.00
Elusimicrobium	0.96	0.84	1.00
GCA-900066225	0.96	0.87	1.00
Akkermansia	0.96	0.89	1.00
UC5-1-2E3	0.96	0.86	1.00
Lachnospiraceae ND3007 group	0.96	0.85	1.00
Selenomonas	0.96	0.82	1.00

Christensenellaceae_uncultured	0.96	1.00	1.00
Candidatus Stoquefichus	0.95	0.83	1.00
Ruminiclostridium 5	0.95	0.83	1.00
Hungatella	0.95	0.91	1.00
Ruminiclostridium	0.95	1.00	1.00
Enterococcus	0.94	0.86	1.00
Marvinbryantia	0.94	0.94	1.00
Flavobacteriaceae_uncultured	0.95	0.92	1.00
Rhodococcus	0.95	0.83	1.00
Roseburia	0.94	0.86	1.00
Ruminococcus 1	0.96	0.84	1.00
Odoribacter	0.95	0.92	1.00
Anaerotruncus	0.95	1.00	1.00
Eubacterium	0.95	0.84	1.00
DTU014_norank	0.95	0.85	1.00
Sharpea	0.95	0.88	1.00
DTU089	0.94	0.83	1.00
Dielma	0.95	0.95	1.00
Solobacterium	0.95	0.87	1.00
Fusobacterium	0.95	1.00	1.00
Coprococcus 2	0.95	0.86	1.00
Lachnospiraceae UCG-008	0.95	0.84	1.00
Hydrogenoanaerobacterium	0.95	0.83	1.00
Acinetobacter	0.95	1.00	1.00
Merdibacter	0.95	0.90	1.00
Defluviitaleaceae UCG-011	0.95	0.88	1.00
Oscillibacter	0.95	0.84	1.00
Mogibacterium	0.94	1.00	1.00
Pelagibacterium	0.94	1.00	1.00

Cloacibacillus	0.94	1.00	1.00
Alloprevotella	0.93	1.00	1.00
Bacteroidales_norank	0.94	0.82	1.00
Prevotella 7	0.93	0.87	1.00
Candidatus Soleaferrea	0.94	0.86	1.00
Mollicutes RF39_norank	0.94	0.83	1.00
Christensenellaceae R-7 group	0.95	1.00	1.00
Howardella	0.95	0.88	1.00
Eisenbergiella	0.96	0.93	1.00
Sellimonas	0.97	0.84	1.00
Acetitomaculum	0.97	0.94	1.00
Microbacterium	0.98	0.85	1.00
Ruminococcaceae_uncultured	1.00	0.93	1.00
Barnesiella	1.00	0.88	1.00
Cardiobacterium	1.00	1.00	1.00
Lachnoanaerobaculum	1.00	1.00	1.00
MBA03_norank	1.00	0.89	1.00
Methylobacterium	1.00	0.90	1.00
Mitochondria_norank	1.00	0.82	1.00
Papillibacter	1.00	0.85	1.00
Stomatobaculum	1.00	0.95	1.00

Supplementary Table 10-5. P values of two-way ANOVA of fecal microbiota incubated with protein digests at the phylum level.

Microbiota	diet	BMI	diet*BMI
Actinobacteria	0.07	0.51	0.25
Bacteroidetes	0.33	0.32	0.74
Cyanobacteria	0.57	0.52	0.89
Euryarchaeota	0.19	0.28	0.79
Firmicutes	0.65	0.38	0.48
Fusobacteria	0.23	0.79	0.37
Lentisphaerae	0.31	0.44	0.48
Proteobacteria	0.29	0.62	0.65
Saccharibacteria	0.24	0.08	0.12
Synergistetes	0.18	0.30	0.38
Tenericutes	0.29	0.33	0.53
Verrucomicrobia	0.11	0.38	0.41
F/B	0.11	0.66	0.52

Supplementary Table 10-6. P values of two-way ANOVA of microbiota incubated with protein digest at the genus level before FDR correction.

Microbiota	diet	BMI	diet*BMI
Bacteroides	0.64	0.96	0.69
Prevotella 9	0.03	0.64	0.76
Escherichia-Shigella	0.02	0.71	0.84
Megasphaera	0.009	0.86	0.92
Faecalibacterium	<0.0001	0.03	0.50
Megamonas	0.005	0.07	0.27
Dialister	0.34	0.99	0.89
Phascolarctobacterium	0.47	0.43	0.91
Parabacteroides	0.11	0.69	0.91
Bifidobacterium	0.28	0.26	0.90
Lachnoclostridium	0.002	0.78	0.72
Alistipes	0.14	0.69	0.93
Sutterella	0.07	0.80	0.86
Blautia	0.03	0.69	0.12
Roseburia	<0.0001	0.95	0.99
Lactobacillus	0.38	0.15	0.13
Streptococcus	0.34	0.13	0.44
Parasutterella	0.74	0.15	0.62
[Ruminococcus] torques group	0.01	0.10	0.62
Veillonella	0.46	0.48	0.92
Fusobacterium	0.58	0.83	0.82
Collinsella	0.003	0.97	0.85
Ruminococcus 2	0.36	0.35	0.84
Enterobacter	0.34	0.49	0.66
Lachnospira	<0.0001	0.51	0.31
[Eubacterium] rectale group	0.004	0.42	0.29

Alloprevotella	0.02	0.59	0.95
Ruminococcaceae UCG-002	0.07	0.14	0.98
Mitsuokella	0.72	0.38	0.91
Lachnospiraceae_uncultured	<0.0001	0.62	0.49
Anaerostipes	0.05	0.99	0.42
[Ruminococcus] gnavus group	0.57	0.49	0.89
Prevotella 2	0.20	0.35	0.84
[Eubacterium] eligens group	0.001	0.44	0.98
Bacteroidales S24-7 group_norank	0.41	0.005	0.89
Subdoligranulum	0.04	0.11	0.27
Paraprevotella	0.37	0.01	0.89
Dorea	0.07	0.16	0.14
Lachnospiraceae NK4A136 group	<0.0001	0.30	0.16
Enterococcus	1.00	0.41	0.86
Fusicatenibacter	<0.0001	0.94	0.99
Desulfovibrio	0.54	0.66	0.84
Holdemanella	0.15	0.73	0.99
[Eubacterium] coprostanoligenes group	<0.0001	0.35	0.35
Bilophila	0.53	0.99	0.43
Ruminococcus 1	0.04	0.49	0.03
Erysipelotrichaceae UCG-003	0.14	0.10	0.30
Lachnospiraceae UCG-004	<0.0001	0.30	0.04
Haemophilus	0.0003	0.68	1.00
[Eubacterium] hallii group	0.25	0.94	0.77
Cronobacter	0.17	0.40	0.99
Ruminococcaceae_uncultured	0.62	0.63	0.77
[Eubacterium] ruminantium group	0.001	0.08	0.04
Ruminococcaceae UCG-014	0.35	0.08	0.77
Butyricicoccus	0.002	0.98	0.86

Peptoclostridium	<0.0001	0.13	0.27
Ruminococcaceae UCG-003	0.12	0.21	0.75
Barnesiella	0.47	0.10	1.00
Christensenellaceae R-7 group	0.15	0.25	0.52
Odoribacter	<0.0001	0.47	1.00
Allisonella	0.06	0.19	0.67
Flavonifractor	0.53	0.21	0.28
Butyricimonas	0.20	0.07	0.82
[Eubacterium] ventriosum group	0.001	0.41	0.55
Coprococcus 3	0.36	0.29	0.58
Ruminococcaceae NK4A214 group	<0.0001	0.93	0.52
Ruminiclostridium 9	0.56	0.94	0.27
Coprococcus 2	0.003	0.46	0.76
Lachnospiraceae UCG-001	<0.0001	0.54	0.61
Coprococcus 1	0.33	0.32	0.35
Clostridium sensu stricto 1	0.22	0.26	0.37
Lactococcus	0.08	0.19	0.14
Lachnospiraceae ND3007 group	0.001	0.83	0.19
Mollicutes RF9_norank	0.26	0.41	0.82
Ruminococcaceae UCG-005	0.002	0.12	0.58
Prevotellaceae NK3B31 group	0.90	0.48	0.96
Catenibacterium	0.76	0.29	0.93
Ruminococcaceae UCG-013	0.003	0.95	0.88
Hungatella	0.54	0.64	0.71
Acidaminococcus	0.74	0.34	0.96
Intestinibacter	0.003	0.59	0.65
Erysipelatoclostridium	0.16	0.30	0.77
Ruminiclostridium 5	0.03	0.55	1.00
Anaerotruncus	0.003	0.82	0.95

[Clostridium] innocuum group	0.89	0.43	0.96
Akkermansia	0.74	0.13	0.98
Coprobacter	0.15	0.27	0.22
[Eubacterium] xylanophilum group	0.03	0.26	0.25
Hafnia-Obesumbacterium	0.24	0.10	0.90
Prevotella 7	0.49	0.70	0.95
Tyzzerella 3	0.13	0.19	0.29
Lachnospiraceae UCG-010	0.90	0.42	0.69
Prevotellaceae_uncultured	0.22	0.34	0.29
Bacteroidales_norank	0.75	0.49	0.32
[Ruminococcus] gauvreauii group	0.03	0.45	0.30
Veillonellaceae_uncultured	0.29	0.52	0.26
Ruminiclostridium 6	0.38	0.26	0.44
Tyzzerella	<0.0001	0.09	0.11
Lachnospiraceae UCG-003	0.53	0.60	0.72
[Eubacterium] fissicatena group	0.58	0.17	0.82
Lachnospiraceae FCS020 group	0.002	0.63	0.72
Weissella	0.26	0.34	0.90
Olsenella	0.39	0.50	0.91
Rhodospirillaceae_uncultured	0.04	0.17	0.40
Tyzzerella 4	0.98	0.48	0.84
Holdemania	0.95	0.69	0.28
Leuconostoc	0.04	0.39	0.74
Eggerthella	0.99	0.36	0.89
Eubacterium	0.08	0.11	0.79
Faecalitalea	0.44	0.55	0.52
Ruminococcaceae UCG-010	0.03	0.44	0.71
Adlercreutzia	0.08	0.04	0.60
Lachnospiraceae_Unclassified	0.27	0.05	0.88

Family XIII AD3011 group	0.09	0.75	0.33
Family XIII UCG-001	0.61	0.57	0.93
Halomonas	0.001	0.55	<0.0001
Lactonifactor	0.18	0.04	0.58
Butyrivibrio	0.62	0.72	0.86
Terrisporobacter	0.02	0.13	0.13
Pyramidobacter	0.81	0.07	0.94
Ruminococcaceae UCG-004	0.002	0.003	0.01
Prevotellaceae UCG-001	0.91	0.74	0.58
Flavobacteriaceae_uncultured	0.68	0.52	1.00
Gastranaerophilales_norank	0.10	0.21	0.40
Oscillospira	0.72	0.48	0.80
Lachnospiraceae UCG-008	0.0002	0.46	0.18
Peptococcus	0.09	0.58	0.99
Brevundimonas	0.97	0.49	1.00
[Bacteroides] pectinophilus group	0.89	0.47	0.96
Candidatus Soleaferrea	0.08	0.97	0.72
Turicibacter	0.03	0.35	0.31
Ruminococcaceae UCG-009	0.01	0.32	0.65
Cloacibacillus	0.65	0.39	0.95
Methanobrevibacter	0.27	0.18	0.73
Intestinimonas	0.60	0.07	0.61
Actinomyces	0.0004	0.42	0.64
Sharpea	0.82	0.49	0.90
Aggregatibacter	0.001	0.51	0.64
Leifsonia	0.23	0.46	0.50
Rhodococcus	0.93	0.47	1.00
Marvinbryantia	0.07	0.12	0.15
Prevotellaceae UCG-004	0.84	0.43	0.27

Senegalimassilia	0.16	0.10	0.13
Christensenellaceae_uncultured	0.70	0.40	0.94
Porphyromonas	0.07	0.64	0.56
Ezakiella	0.15	0.32	0.31
Coriobacteriaceae_uncultured	0.10	0.70	0.08
Rikenellaceae RC9 gut group	0.24	0.23	0.22
Gemella	0.07	0.60	0.55
Pseudobutyrivibrio	0.08	0.11	0.58
Pseudomonas	0.19	0.86	0.67
Enterorhabdus	0.97	0.03	1.00
Ruminococcaceae UCG-008	0.40	0.25	0.99
Oxalobacter	0.79	0.69	0.17
Christensenella	0.48	0.11	0.82
Sellimonas	0.56	0.10	0.97
Howardella	0.85	0.59	0.78
Dielma	0.56	0.92	0.62
Anaerofustis	0.12	0.13	0.67
Slackia	0.34	0.26	0.55
Microbacterium	0.28	0.62	0.53
Victivallis	0.49	0.21	0.68
Comamonas	0.11	0.22	0.13
Chloroplast_norank	0.10	0.004	0.37
Prevotella 6	0.38	0.37	0.46
Defluviitaleaceae UCG-011	0.85	0.45	0.97
Faecalicoccus	0.35	0.15	0.37
[Eubacterium] brachy group	0.93	0.72	0.49
Saccharibacteria_norank	0.02	0.55	0.16
Clostridiales vadinBB60 group_norank	0.02	0.15	0.15
Prevotella	0.22	0.50	0.55

Campylobacter	0.57	0.23	0.33
Parvimonas	0.28	0.16	0.67
Lachnospiraceae NC2004 group	0.001	0.84	0.17
Aliihoeflea	0.35	0.90	0.45
Eisenbergiella	0.44	0.23	0.89
Pelagibacterium	0.008	0.74	0.14
Acinetobacter	0.76	0.73	0.63
Peptococcaceae_uncultured	0.27	0.73	0.69
Rothia	0.03	0.31	0.72
Candidatus Stoquefichus	0.19	0.18	0.09
Oribacterium	0.09	0.40	0.21
Ruminiclostridium	0.41	0.23	0.33
Morganella	0.48	0.10	0.48
Oscillibacter	0.15	0.67	0.35
Hydrogenoanaerobacterium	0.55	0.35	0.83
Erysipelotrichaceae UCG-004	0.20	0.05	0.41
Gordonibacter	0.55	0.42	0.95
vadinBE97_norank	0.93	0.60	0.94
[Eubacterium] nodatum group	0.81	0.04	0.99
Peptoniphilus	0.42	0.13	0.49
Succinivibrionaceae_uncultured	0.26	0.20	0.24
Aeromonas	0.04	0.19	0.16
Parvibacter	0.32	0.35	0.55
Staphylococcus	0.49	0.09	0.88
Peptostreptococcus	0.04	0.29	0.46
Mogibacterium	0.37	0.70	0.37
Epulopiscium	0.73	0.62	0.82
Paraclostridium	0.69	0.11	0.95
Methanosphaera	0.39	0.44	0.93

Anaerofilum	0.14	0.65	0.55
Acetitomaculum	0.29	0.32	0.51
Proteus	0.73	0.03	0.74
Solobacterium	0.001	0.91	0.98
Nesterenkonia	0.59	0.44	0.30
Abiotrophia	0.95	0.69	0.22
Mitochondria_norank	0.26	0.35	0.44
Neisseria	0.15	0.64	0.77
Opitutae vadinHA64_norank	0.82	0.43	0.96
Sarcina	0.75	0.57	0.96
Lachnoanaerobaculum	0.61	0.49	0.55
Gelria	0.43	0.32	0.54
Corynebacterium	0.59	0.54	0.29
Papillibacter	0.11	0.31	0.13
Bacillus	0.12	0.16	0.04
Erysipelotrichaceae UCG-006	0.23	0.24	0.21
Finegoldia	0.04	0.67	0.49
Johnsonella	0.30	0.55	0.48
Ruminococcaceae UCG-011	0.78	0.76	0.58
Stomatobaculum	0.35	0.58	0.67
Lachnospiraceae NK3A20 group	0.23	0.24	0.21
Rikenella	0.65	0.66	0.79
Actinomycetaceae_uncultured	0.002	0.08	0.04
Anaerococcus	0.04	0.23	0.20
Sporobacter	0.10	0.58	0.69
Phyllobacteriaceae_uncultured	0.89	0.42	0.96
Murdochiella	0.99	0.66	0.79
Tenacibaculum	0.23	0.24	0.21

Supplementary Table 10-7. P values of ANOVA of microbiota incubated with protein digest at the genus level after FDR correction.

Microbiota	diet	BMI	diet*BMI
Faecalibacterium	0.02	0.68	1.00
Roseburia	0.01	0.46	1.00
Lachnospira	0.008	0.38	1.00
Lachnospiraceae_uncultured	0.006	0.68	1.00
Lachnospiraceae NK4A136 group	0.005	1.00	1.00
Fusicatenibacter	0.004	1.00	1.00
[Eubacterium] coprostanoligenes group	0.003	1.00	1.00
Lachnospiraceae UCG-004	0.003	1.00	1.00
Peptoclostridium	0.003	1.00	1.00
Odoribacter	0.002	1.00	1.00
Ruminococcaceae NK4A214 group	0.002	1.00	1.00
Lachnospiraceae UCG-001	0.002	1.00	1.00
Tyzzerella	0.002	1.00	1.00
Lachnospiraceae UCG-008	0.003	1.00	1.00
Haemophilus	0.005	1.00	1.00
Actinomyces	0.006	1.00	1.00
[Eubacterium] eligens group	0.01	1.00	1.00
[Eubacterium] ruminantium group	0.01	1.00	1.00
[Eubacterium] ventriosum group	0.01	1.00	1.00
Lachnospiraceae ND3007 group	0.01	1.00	1.00
Halomonas	0.01	1.00	0.02
Aggregatibacter	0.01	1.00	1.00
Lachnospiraceae NC2004 group	0.001	0.97	1.00
Solobacterium	0.01	0.94	1.00
Lachnoclostridium	0.02	0.92	1.00
Butyricicoccus	0.02	0.89	1.00

Ruminococcaceae UCG-005	0.02	0.86	1.00
Lachnospiraceae FCS020 group	0.02	0.84	1.00
Ruminococcaceae UCG-004	0.02	0.83	1.00
Actinomycetaceae_uncultured	0.02	0.84	1.00
Collinsella	0.02	0.82	1.00
Coprococcus 2	0.02	0.81	1.00
Ruminococcaceae UCG-013	0.02	0.79	1.00
Intestinibacter	0.02	0.78	1.00
Anaerotruncus	0.02	0.77	1.00
[Eubacterium] rectale group	0.03	0.79	1.00
Megamonas	0.03	0.78	1.00
Pelagibacterium	0.04	0.78	1.00
Megasphaera	0.05	0.77	1.00
[Ruminococcus] torques group	0.06	0.76	1.00
Ruminococcaceae UCG-009	0.07	0.75	1.00
Terrisporobacter	0.10	0.78	1.00
Escherichia-Shigella	0.11	0.77	1.00
Saccharibacteria_norank	0.11	0.76	1.00
Alloprevotella	0.12	0.77	1.00
Clostridiales vadinBB60 group_norank	0.12	0.75	1.00
Blautia	0.12	0.77	1.00
Ruminococcaceae UCG-010	0.12	0.77	1.00
Prevotella 9	0.14	0.76	1.00
[Eubacterium] xylanophilum group	0.14	0.77	1.00
[Ruminococcus] gauvreauii group	0.13	0.76	1.00
Turicibacter	0.14	0.79	1.00
Ruminiclostridium 5	0.15	0.79	1.00
Rothia	0.14	0.79	1.00
Subdoligranulum	0.15	0.77	1.00

Aeromonas	0.14	0.77	1.00
Peptostreptococcus	0.14	0.78	1.00
Leuconostoc	0.15	0.78	1.00
Finegoldia	0.17	0.79	1.00
Anaerococcus	0.18	0.79	1.00
Ruminococcus 1	0.18	0.80	1.00
Rhodospirillaceae_uncultured	0.18	0.78	1.00
Anaerostipes	0.19	0.78	1.00
Allisonella	0.20	0.81	1.00
Sutterella	0.23	0.81	1.00
Dorea	0.23	0.80	1.00
Marvinbryantia	0.23	0.79	1.00
Porphyromonas	0.23	0.79	1.00
Ruminococcaceae UCG-002	0.23	0.79	1.00
Gemella	0.24	0.78	1.00
Pseudobutyrivibrio	0.24	0.76	1.00
Adlercreutzia	0.24	0.79	1.00
Eubacterium	0.24	0.79	1.00
Candidatus Soleaferrea	0.24	0.79	1.00
Lactococcus	0.24	0.78	1.00
Oribacterium	0.25	0.77	1.00
Family XIII AD3011 group	0.26	0.76	1.00
Peptococcus	0.27	0.77	1.00
Chloroplast_norank	0.28	0.79	1.00
Gastranaerophilales_norank	0.28	0.82	1.00
Sporobacter	0.29	0.82	1.00
Coriobacteriaceae_uncultured	0.29	0.82	1.00
Parabacteroides	0.29	0.83	1.00
Comamonas	0.30	0.82	1.00

Papillibacter	0.31	0.81	1.00
Ruminococcaceae UCG-003	0.31	0.82	1.00
Bacillus	0.31	0.82	1.00
Anaerofustis	0.32	0.82	1.00
Tyzzerella 3	0.33	0.82	1.00
Anaerofilum	0.35	0.81	1.00
Erysipelotrichaceae UCG-003	0.35	0.81	1.00
Alistipes	0.36	0.80	1.00
Christensenellaceae R-7 group	0.36	0.83	1.00
Coprobacter	0.35	0.83	1.00
Oscillibacter	0.35	0.82	1.00
Holdemanella	0.36	0.82	1.00
Ezakiella	0.36	0.82	1.00
Neisseria	0.36	0.81	1.00
Erysipelatoclostridium	0.36	0.81	1.00
Senegalimassilia	0.36	0.80	1.00
Cronobacter	0.39	0.81	1.00
Lactonifactor	0.41	0.81	1.00
Candidatus Stoquefichus	0.41	0.82	1.00
Pseudomonas	0.42	0.83	1.00
Butyricimonas	0.43	0.85	1.00
Erysipelotrichaceae UCG-004	0.43	0.85	1.00
Prevotella 2	0.43	0.85	1.00
Prevotellaceae_uncultured	0.46	0.85	1.00
Prevotella	0.46	0.84	1.00
Clostridium sensu stricto 1	0.46	0.84	1.00
Leifsonia	0.47	0.85	1.00
Erysipelotrichaceae UCG-006	0.48	0.84	1.00
Lachnospiraceae NK3A20 group	0.47	0.84	1.00

Tenacibaculum	0.47	0.83	1.00
Hafnia-Obesumbacterium	0.47	0.83	1.00
Rikenellaceae RC9 gut group	0.47	0.83	1.00
[Eubacterium] hallii group	0.48	0.82	1.00
Succinivibrionaceae_uncultured	0.50	0.82	1.00
Weissella	0.50	0.82	1.00
Mollicutes RF9_norank	0.50	0.82	1.00
Mitochondria_norank	0.50	0.82	1.00
Lachnospiraceae_Unclassified	0.50	0.81	1.00
Methanobrevibacter	0.50	0.81	1.00
Peptococcaceae_uncultured	0.50	0.81	1.00
Microbacterium	0.51	0.81	1.00
Bifidobacterium	0.51	0.81	1.00
Parvimonas	0.51	0.81	1.00
Veillonellaceae_uncultured	0.51	0.81	1.00
Acetitomaculum	0.52	0.82	1.00
Johnsonella	0.52	0.81	1.00
Parvibacter	0.56	0.81	1.00
Coprococcus 1	0.58	0.81	1.00
Streptococcus	0.57	0.81	1.00
Slackia	0.57	0.81	1.00
Enterobacter	0.57	0.81	1.00
Dialister	0.58	0.81	1.00
Ruminococcaceae UCG-014	0.57	0.81	1.00
Stomatobaculum	0.57	0.80	1.00
Faecalicoccus	0.58	0.80	1.00
Aliihoeflea	0.58	0.79	1.00
Ruminococcus 2	0.57	0.79	1.00
Coprococcus 3	0.58	0.78	1.00

Mogibacterium	0.59	0.78	1.00
Paraprevotella	0.59	0.78	1.00
Lactobacillus	0.59	0.79	1.00
Prevotella 6	0.59	0.78	1.00
Ruminiclostridium 6	0.59	0.78	1.00
Methanosphaera	0.60	0.79	1.00
Olsenella	0.60	0.79	1.00
Ruminococcaceae UCG-008	0.61	0.79	1.00
Bacteroidales S24-7 group_norank	0.62	0.81	1.00
Ruminiclostridium	0.62	0.81	1.00
Peptoniphilus	0.62	0.82	1.00
Gelria	0.63	0.81	1.00
Faecalitalea	0.64	0.81	1.00
Eisenbergiella	0.65	0.81	1.00
Veillonella	0.66	0.80	1.00
Phascolarctobacterium	0.67	0.82	1.00
Barnesiella	0.68	0.82	1.00
Morganella	0.68	0.82	1.00
Christensenella	0.68	0.82	1.00
Staphylococcus	0.68	0.82	1.00
Victivallis	0.68	0.83	1.00
Prevotella 7	0.68	0.82	1.00
Bilophila	0.73	0.82	1.00
Flavonifractor	0.72	0.82	1.00
Lachnospiraceae UCG-003	0.72	0.82	1.00
Desulfovibrio	0.73	0.82	1.00
Hungatella	0.73	0.83	1.00
Gordonibacter	0.74	0.83	1.00
Hydrogenoanaerobacterium	0.74	0.82	1.00

Sellimonas	0.736	0.83	1.00
Ruminiclostridium 9	0.743	0.83	1.00
Dielma	0.739	0.83	1.00
[Ruminococcus] gnavus group	0.739	0.83	1.00
Campylobacter	0.737	0.83	1.00
[Eubacterium] fissicatena group	0.745	0.83	1.00
Fusobacterium	0.748	0.83	1.00
Corynebacterium	0.746	0.83	1.00
Nesterenkonia	0.744	0.83	1.00
Intestinimonas	0.750	0.83	1.00
Lachnoanaerobaculum	0.758	0.84	1.00
Family XIII UCG-001	0.755	0.83	1.00
Ruminococcaceae_uncultured	0.771	0.85	1.00
Butyrivibrio	0.768	0.84	1.00
Bacteroides	0.785	0.84	1.00
Cloacibacillus	0.789	0.84	1.00
Rikenella	0.791	0.83	1.00
Flavobacteriaceae_uncultured	0.820	0.83	1.00
Paraclostridium	0.826	0.83	1.00
Christensenellaceae_uncultured	0.832	0.83	1.00
Oscillospira	0.857	0.83	1.00
Mitsuokella	0.854	0.83	1.00
Epulopiscium	0.856	0.83	1.00
Proteus	0.858	0.84	1.00
Akkermansia	0.861	0.84	1.00
Parasutterella	0.858	0.84	1.00
Acidaminococcus	0.853	0.84	1.00
Bacteroidales_norank	0.860	0.83	1.00
Sarcina	0.860	0.84	1.00

Acinetobacter	0.86	0.84	1.00
Catenibacterium	0.86	0.85	1.00
Ruminococcaceae UCG-011	0.87	0.86	1.00
Oxalobacter	0.89	0.87	1.00
[Eubacterium] nodatum group	0.90	0.89	1.00
Pyramidobacter	0.90	0.91	1.00
Sharpea	0.90	0.91	1.00
Opitutae vadinHA64_norank	0.90	0.91	1.00
Prevotellaceae UCG-004	0.92	0.91	1.00
Defluviitaleaceae UCG-011	0.92	0.94	1.00
Howardella	0.92	0.93	1.00
Phyllobacteriaceae_uncultured	0.95	0.96	1.00
[Bacteroides] pectinophilus group	0.96	0.97	1.00
[Clostridium] innocuum group	0.95	0.98	1.00
Lachnospiraceae UCG-010	0.95	0.99	1.00
Prevotellaceae NK3B31 group	0.95	0.99	1.00
Prevotellaceae UCG-001	0.96	0.99	1.00
vadinBE97_norank	0.97	0.99	1.00
[Eubacterium] brachy group	0.96	0.99	1.00
Rhodococcus	0.97	0.99	1.00
Holdemania	0.98	0.99	1.00
Abiotrophia	0.98	0.99	1.00
Brevundimonas	0.99	0.99	1.00
Enterorhabdus	0.99	1.00	1.00
Tyzzerella 4	0.99	1.00	1.00
Eggerthella	0.99	1.00	1.00
Murdochiella	0.99	1.00	1.00
Enterococcus	1.00	0.35	1.00

Supplementary Table 10-8. P values of two-way repeated measures ANOVA of fecal metabolites before FDR correction.

Metabolites	diet	BMI	diet*BMI
(4Z,7Z,10Z,13Z,16Z,19Z)-Docosahexaenoic acid	0.19	0.19	0.39
(5α,6α)-7,8-Didehydro-4,5-epoxy-3-methoxy -17-methylmorphinan-6-ol	<0.0001	0.07	0.95
(8Z,11Z,14Z)-Icosatrienoic acid	0.19	0.79	0.66
(S)-2-Acetolactate	0.001	0.27	0.90
(S)-3-Hydroxy-3-methylglutaryl-CoA	<0.0001	0.82	0.14
1,2-Dihydroxy-3,4-epoxy-1,2,3,4 -tetrahydronaphthalene	<0.0001	<0.0001	0.03
11H-14,15-EETA	0.001	0.25	0.14
12(13)-EpOME	0.009	0.43	0.16
12-oxo-20-trihydroxy-leukotriene B4	<0.0001	0.08	0.67
13'-carboxy-γ-tocopherol	<0.0001	0.33	0.44
17α, 21-Dihydroxypregnenolone	0.44	0.48	0.81
18-Oxocortisol	0.03	0.55	0.46
20-carboxy-leukotriene-B4	<0.0001	0.28	0.37
20-dihydroxyleukotriene B4	0.96	0.13	0.46
20-Hydroxyleukotriene E4	<0.0001	0.04	0.41
24-oxo-1α,25-dihydroxyvitamin D3	0.79	0.60	0.71
3,4-Dihydroxyphenylacetaldehyde	0.02	0.30	0.56
3-Dehydrosphinganine	0.003	0.38	0.30
3-Hexaprenyl-4,5-dihydroxybenzoate	0.007	0.37	0.66
3-Hydroxycarbamazepine	<0.0001	0.47	0.68
3-Methoxy-4-hydroxyphenylacetaldehyde	0.001	0.01	0.39
3-Methoxy-4-hydroxyphenylglycolaldehyde	<0.0001	0.40	0.40
3α,21-Dihydroxy-5β-pregnane-11,20-dione	0.44	0.48	0.81
3α,7α,12α-trihydroxy-5β-cholanate	0.0004	0.008	0.02

3α,7α-Dihydroxy-5β-cholestane	0.03	0.01	0.29
3β-Hydroxyandrost-5-en-17-one 3-sulfate	<0.0001	0.98	0.29
4-Coumarate	0.26	0.94	0.77
4-Hydroxyphenylacetaldehyde	0.16	0.14	0.49
5-Dehydroepisterol	<0.0001	0.15	0.02
7'-carboxy-α-chromanol	0.003	0.71	0.64
9(s)-hydroperoxy-10(e),12(Z),15(Z)-octadecatrieno	0.003	0.13	0.12
9'-carboxy-γ-chromanol	<0.0001	0.13	0.37
9'-carboxy-γ-tocotrienol	0.005	0.37	0.80
Acetic acid	0.22	0.94	0.30
Acetoin	0.04	0.19	0.03
acetylcarnosine	<0.0001	0.41	0.78
Adipic acid	0.17	0.83	0.27
Adrenosterone	<0.0001	0.06	0.15
Alanine	0.07	0.63	0.69
Aminoacetone	<0.0001	0.29	<0.0001
Aminoadipate	0.35	0.59	0.26
Anandamide	0.005	0.99	0.89
Arabitol	0.05	0.06	0.45
arachidyl carnitine	0.11	0.42	0.76
Aspartate	0.03	0.04	0.48
Azelaic acid	0.27	0.07	0.07
Butyrate	0.04	0.72	0.09
Butyric acid	0.28	0.88	0.28
Campest-4-en-3β-ol	<0.0001	0.37	0.04
Carnitine	0.07	0.23	0.24
choline	0.39	0.47	0.29
Chondroitin	<0.0001	0.03	0.02
Citruline	0.29	0.08	0.37

Cytidine	<0.0001	0.95	0.79
Demethylcitalopram	<0.0001	0.03	0.24
Deoxyguanosine	0.001	0.004	0.56
Deoxyuridine	0.002	0.47	0.19
Didemethylcitalopram	<0.0001	0.17	0.04
Dihydrolipoamide	0.0004	0.63	0.44
Erythrose	0.83	0.44	0.28
Flavin mononucleotide	<0.0001	0.003	<0.0001
FMNH	<0.0001	0.003	<0.0001
FMNH2	0.006	0.83	0.07
Fumaric acid	0.06	0.09	0.36
Geranylgeranyl diphosphate	0.004	0.41	0.87
Gluconic acid	0.04	0.27	0.59
Glucose	0.13	0.04	0.09
Glucose-1-phosphate	0.24	0.007	0.37
Glutamate	0.23	0.98	0.28
Glycerol	0.03	0.38	0.19
Glycine	0.04	0.28	0.43
Glycocholic acid	0.03	0.38	0.33
hexacosanoyl carnitine	<0.0001	0.0003	0.43
Hexadecanoic acid	0.002	0.99	0.99
Hypoxanthine	0.04	0.15	0.57
Imidazole-4-acetaldehyde	0.002	0.007	0.06
Indole	0.06	0.09	0.12
Irinotecan	0.002	0.02	0.24
Isobutyric acid	0.28	0.28	0.69
Isoleucine	0.001	0.43	0.31
Isovaleric acid	0.48	0.27	0.50
Kynurenine	0.07	0.22	0.57

Lactic acid	0.27	0.03	0.03
Leucine	0.03	0.39	0.26
L-Normetanephrine	0.02	0.26	0.73
lysine	0.002	0.59	0.90
L-α-Acetyl-N,N-dinormethadol	<0.0001	0.09	0.31
Maltose	0.04	0.08	0.61
Mannose	0.07	0.17	0.29
Mesaconate	0.01	0.49	0.62
Methionine	0.02	0.50	0.03
Methylimidazoleacetic acid	<0.0001	0.38	0.73
Monopalmitin	0.14	0.52	0.28
N6,N6,N6-Trimethyl-L-lysine	<0.0001	0.008	0.02
N-Acetyllactosamine	0.002	0.55	0.71
N-Acetylputrescine	0.001	0.43	0.31
Nicotinic acid	<0.0001	0.06	0.02
Norcodeine	0.21	0.45	0.62
p-cresol	0.15	0.53	0.33
Phosphorylcholine	<0.0001	0.07	0.07
Phylloquinone	0.06	0.22	0.43
Phytosphingosine	0.0008	0.40	0.10
Pimelic acid	0.18	0.66	0.20
Proline	0.03	0.01	0.98
Propionate	0.16	0.40	0.55
Prostaglandin G2	<0.0001	0.74	0.24
Psychosine	0.001	0.60	0.29
putrescine	0.04	0.18	0.92
Pyruvic acid	0.006	0.49	0.84
Ribose	0.06	<0.0001	0.38
Saccharopine	0.002	0.33	0.89

Skatole	0.04	0.20	0.29
Sorbitol	0.28	0.02	0.62
Sphinganine	<0.0001	0.14	0.20
Sphingosine	0.11	0.39	0.03
Sphingosine 1-phosphate	<0.0001	0.70	0.85
Stilbene oxide	0.0002	0.14	0.02
Succinate	0.28	0.26	0.73
Sulfatide	0.14	0.90	0.44
Theonic acid	0.09	0.38	0.19
Theonine	0.03	0.27	0.92
Thioctic acid amide	<0.0001	0.92	0.49
Threitol	0.48	0.26	0.37
Threose	0.07	0.08	0.72
Thymine	0.001	0.96	0.53
Thymine	0.17	0.04	0.26
Ubiquinol	0.23	0.04	0.15
Uracil	0.03	0.03	0.47
Uridine	0.03	0.29	0.62
Valeric acid	0.16	0.91	0.29
Valine	0.08	0.29	0.76
Vitamin K1 epoxide	<0.0001	0.27	0.65
Xanthine	0.07	0.83	0.83
Xylose	0.03	0.29	0.19
α-Tocotrienol	0.003	0.05	0.02
β-Aminopropion aldehyde	<0.0001	0.37	0.0002

Supplementary Table 10-9. P values of two-way repeated measures ANOVA of fecal metabolites after FDR correction.

Metabolites	diet	BMI	Diet*BMI
hexacosanoyl carnitine	0.01	0.01	0.77
Demethylcitalopram	0.07	0.24	0.29
3-Hydroxycarbamazepine	0.04	0.65	0.87
Didemethylcitalopram	0.03	0.50	0.29
Aminoacetone	0.03	0.58	0.01
Sphinganine	0.02	0.44	0.71
Methylimidazoleacetic acid	0.02	0.64	0.87
20-carboxy-leukotriene-B4	0.02	0.57	0.74
N6,N6,N6-Trimethyl-L-lysine	0.02	0.11	0.34
1,2-Dihydroxy-3,4-epoxy-1,2,3,4-tetrahydronaphthalene	0.01	0.01	0.31
Campest-4-en-3β-ol	0.01	0.67	0.31
5-Dehydroepisterol	0.01	0.44	0.39
Adrenosterone	0.01	0.30	0.67
12-oxo-20-trihydroxy-leukotriene B4	0.01	0.33	0.86
20-Hydroxyleukotriene E4	0.009	0.24	0.74
L-α-Acetyl-N,N-dinormethadol	0.008	0.34	0.68
Vitamin K1 epoxide	0.008	0.60	0.86
β-Aminopropion aldehyde	0.008	0.66	0.03
(5α,6α)-7,8-Didehydro-4,5-epoxy-3-methoxy-17-methyl morphinan-6-ol	0.007	0.31	0.96
acetylcarnosine	0.007	0.63	0.89
Sphingosine 1-phosphate	0.007	0.82	0.92
3β-Hydroxyandrost-5-en-17-one 3-sulfate	0.007	0.99	0.70
Chondroitin	0.006	0.22	0.27
3-Methoxy-4-hydroxyphenylglycolaldehyde	0.006	0.62	0.73
13'-carboxy-γ-tocopherol	0.006	0.61	0.74

Prostaglandin G2	0.006	0.85	0.80
Cytidine	0.006	0.99	0.89
FMNH	0.005	0.10	0.07
Nicotinic acid	0.005	0.30	0.29
Flavin mononucleotide	0.005	0.08	0.05
Phosphorylcholine	0.005	0.31	0.46
Thioctic acid amide	0.005	0.98	0.76
9'-carboxy-γ-chromanol	0.005	0.44	0.73
(S)-3-Hydroxy-3-methylglutaryl-CoA	0.005	0.92	0.68
Stilbene oxide	0.0007	0.43	0.46
3α,7α,12α-trihydroxy-5β-cholanate	0.002	0.12	0.39
Dihydrolipoamide	0.002	0.76	0.74
Phytosphingosine	0.003	0.62	0.53
N-Acetylputrescine	0.004	0.63	0.69
Psychosine	0.003	0.73	0.74
Isoleucine	0.003	0.62	0.68
3-Methoxy-4-hydroxyphenylacetaldehyde	0.003	0.14	0.73
11H-14,15-EETA	0.003	0.62	0.66
Deoxyguanosine	0.003	0.09	0.82
Thymine	0.003	0.25	0.79
(S)-2-Acetolactate	0.003	0.60	0.94
lysine	0.006	0.73	0.94
Imidazole-4-acetaldehyde	0.006	0.14	0.42
Hexadecanoic acid	0.006	0.99	0.99
Deoxyuridine	0.005	0.65	0.76
Saccharopine	0.005	0.61	0.94
Irinotecan	0.005	0.15	0.79
N-Acetyllactosamine	0.005	0.70	0.88
3-Dehydrosphinganine	0.008	0.63	0.70

9(s)-hydroperoxy-10(e),12(Z),15(Z)-octadecatrieno	0.007	0.43	0.61
α-Tocotrienol	0.007	0.28	0.30
7'-carboxy-α-chromanol	0.007	0.82	0.85
Geranylgeranyl diphosphate	0.009	0.63	0.94
Anandamide	0.01	1.00	0.95
9'-carboxy-γ-tocotrienol	0.01	0.67	0.90
FMNH2	0.01	0.91	0.44
Pyruvic acid	0.01	0.64	0.92
3-Hexaprenyl-4,5-dihydroxybenzoate	0.02	0.65	0.86
12(13)-EpOME	0.02	0.63	0.68
Mesaconate	0.03	0.65	0.84
3,4-Dihydroxyphenylacetaldehyde	0.03	0.57	0.82
Methionine	0.03	0.65	0.25
L-Normetanephrine	0.04	0.61	0.86
3α,7α-Dihydroxy-5β-cholestane	0.05	0.15	0.73
Proline	0.05	0.15	0.99
Uracil	0.05	0.22	0.76
xylose	0.05	0.56	0.76
Theonine	0.05	0.60	0.94
Glycocholic acid	0.05	0.65	0.71
18-Oxocortisol	0.05	0.70	0.75
Leucine	0.05	0.62	0.80
Aspartate	0.05	0.24	0.76
Glycerol	0.05	0.63	0.74
Uridine	0.06	0.58	0.84
Acetoin	0.06	0.51	0.27
putrescine	0.06	0.49	0.95
Skatole	0.07	0.53	0.72
Hypoxanthine	0.08	0.44	0.82

Glycine	0.08	0.58	0.76
Maltose	0.08	0.33	0.86
Butyrate	0.08	0.83	0.52
Gluconic acid	0.08	0.58	0.84
Arabitol	0.08	0.75	0.75
Ribose	0.09	0.07	0.72
Phylloquinone	0.09	0.56	0.75
Indole	0.09	0.33	0.59
Fumaric acid	0.09	0.33	0.75
Carnitine	0.10	0.56	0.82
Kynurenine	0.10	0.56	0.83
Xanthine	0.11	0.91	0.91
Alanine	0.10	0.75	0.86
Mannose	0.10	0.49	0.76
Threose	0.10	0.33	0.88
Valine	0.11	0.57	0.89
Theonic acid	0.12	0.65	0.72
arachidyl carnitine	0.15	0.63	0.89
Sphingosine	0.15	0.62	0.26
Glucose	0.17	0.25	0.52
Sulfatide	0.18	0.97	0.75
p-cresol	0.19	0.68	0.70
Monopalmitin	0.19	0.67	0.81
4-Hydroxyphenylacetaldehyde	0.20	0.44	0.76
Propionate	0.20	0.63	0.82
Valeric acid	0.21	0.97	0.71
Adipic acid	0.21	0.92	0.81
Thymine	0.21	0.99	0.80
Pimelic acid	0.22	0.78	0.72

(8Z,11Z,14Z)-Icosatrienoic acid	0.23	0.90	0.87
(4Z,7Z,10Z,13Z,16Z,19Z)-Docosahexaenoic acid	0.23	0.52	0.73
Norcodeine	0.25	0.64	0.85
Acetic acid	0.25	0.98	0.70
Ubiquinol	0.26	0.25	0.65
Glutamate	0.27	0.99	0.79
Glucose-1-phosphate	0.28	0.12	0.75
4-Coumarate	0.29	0.99	0.89
Azelaic acid	0.31	0.30	0.46
Lactic acid	0.31	0.22	0.28
Sorbitol	0.31	0.16	0.86
Isobutyric acid	0.31	0.59	0.86
Succinate	0.31	0.60	0.87
Butyric acid	0.31	0.95	0.77
Citruline	0.31	0.33	0.76
Aminoadipate	0.37	0.73	0.82
choline	0.41	0.65	0.75
17α, 21-Dihydroxypregnenolone	0.46	0.66	0.90
3α,21-Dihydroxy-5β-pregnane-11,20-dione	0.45	0.65	0.89
Threitol	0.49	0.61	0.72
Isovaleric acid	0.49	0.59	0.76
24-oxo-1α,25-dihydroxyvitamin D3	0.80	0.74	0.87
Erythrose	0.83	0.63	0.80
20-dihydroxyleukotriene B4	0.96	0.44	0.75

Supplementary Table 10-10. P values of two-way repeated measures ANOVA of fecal proteomics before FDR correction.

	diet	BMI	diet*BMI
P0DOY3	0.61	0.03	0.72
A2ASS6	0.23	0.07	0.16
Q2RLT8	0.48	0.02	0.53
A6KXL2	0.007	<0.0001	0.003
Q8AAW1	0.10	0.0004	0.04
A6L5A6	0.14	0.003	0.07
A6LFQ4	0.03	0.002	0.01
A8K7I4	0.0002	<0.0001	0.92
Q9PE76	0.56	0.64	0.64
B2UYT8	0.35	0.03	0.53
B5YFN3	0.47	0.51	0.51
C4ZBL1	0.10	0.03	0.04
P86210	0.13	<0.0001	0.09
O22317	0.37	<0.0001	0.69
O43451	0.72	<0.0001	0.80
O73860	0.49	0.01	0.55
P60052	0.99	0.0003	0.90
Q9XSC6	0.008	0.002	0.003
P00688	0.28	0.12	0.23
P00761	0.52	0.0002	0.65
P00813	0.56	0.64	0.64
P01009	0.51	0.0002	0.57
P01012	0.78	0.02	0.91
P01591	0.01	<0.0001	0.0004
P01833	0.02	<0.0001	0.16
P0DOX7	0.59	<0.0001	0.59

P0DOX6	0.83	0.02	0.94
P01876	0.64	0.0005	0.75
P0DOX2	0.0003	<0.0001	<0.0001
P02763	0.57	0.33	0.68
P02768	0.72	0.12	0.84
P04054	0.70	0.0002	0.88
P04118	0.87	0.35	0.97
P04264	0.002	0.001	0.46
P04746	0.11	0.01	0.02
P06702	0.003	0.001	0.84
P06731	0.35	0.04	0.33
P06870	0.07	0.004	0.17
P07478	0.50	<0.0001	0.65
P08217	0.55	<0.0001	0.21
Q5RE69	0.39	<0.0001	0.65
P08861	0.62	<0.0001	0.93
P09093	0.20	<0.0001	0.36
P09923	0.34	<0.0001	0.15
P84589	0.18	0.0004	0.22
P10994	0.54	0.58	0.61
P12821	0.90	0.001	0.98
P13538	0.84	0.001	0.95
Q02897	0.24	0.11	0.17
P15085	0.004	0.0002	0.0005
P15086	0.08	<0.0001	0.01
P15144	0.27	<0.0001	0.17
P16233	0.23	0.08	0.25
Q6GPI1	0.03	0.004	0.22
P19961	0.02	<0.0001	0.02

P20111	0.51	0.56	0.56
P20758	0.49	<0.0001	0.62
P27487	0.69	<0.0001	0.85
P48052	0.71	0.01	0.82
P78010	0.56	0.64	0.64
Q9BE39	0.44	0.18	0.46
Q02817	0.18	<0.0001	0.61
Q03403	0.56	0.64	0.64
Q5RDA4	0.12	<0.0001	0.57
Q13228	0.50	<0.0001	0.56
Q16820	0.82	0.19	0.94
Q1WUJ3	0.26	0.06	0.55
Q47VD0	0.20	<0.0001	0.48
Q5KR49	0.04	0.001	0.03
Q64MV4	0.12	<0.0001	0.28
Q8A9M2	0.09	<0.0001	0.07
Q6UWV6	0.11	<0.0001	0.51
Q86UP6	0.24	<0.0001	0.28
Q8A1A2	0.09	0.005	0.04
Q8A414	0.32	0.004	0.28
Q8A6N4	0.06	0.06	0.06
Q9BYE9	0.11	0.02	0.33
Q9CQY9	0.54	0.62	0.62
Q9H3R2	0.53	0.01	0.61
Q9NR71	0.20	0.14	0.18
Q9TV62	0.11	<0.0001	0.06
Q9UQQ1	0.49	0.26	0.54
Q9Y6R7	0.44	<0.0001	0.20
A9KQ65	0.26	0.03	0.29

B3H1D9	0.04	0.03	0.02
A5A6M6	0.23	<0.0001	0.24
A6L792	0.35	0.05	0.43
A6LGR3	0.21	0.23	0.23
Q7MWI7	0.75	<0.0001	0.90
P0DOX8	0.66	<0.0001	0.83
C4Z1J4	0.22	0.19	0.23
C4ZF71	0.21	0.13	0.22
Q0VCY0	0.03	<0.0001	0.01
O43895	0.71	0.001	0.90
Q9TSX8	0.23	0.0003	0.25
P05208	0.21	0.23	0.23
P01721	0.21	0.23	0.23
P01887	0.21	0.23	0.23
P02565	0.37	<0.0001	0.46
P10246	0.0003	0.0002	<0.0001
P02766	0.44	<0.0001	0.56
Q5NVH5	0.23	0.21	0.25
P02788	0.15	<0.0001	0.14
Q5R538	0.15	0.14	0.14
P05451	0.04	0.01	0.02
P08454	0.24	0.20	0.26
Q9GLN7	0.32	<0.0001	0.39
P12883	0.0005	0.0002	0.81
P12955	0.03	0.04	0.01
P14410	0.04	0.0002	0.02
P18600	0.04	0.0001	0.02
P35527	0.13	0.02	0.11
Q9WUH4	0.12	0.10	0.10

P98088	0.001	<0.0001	0.0001
Q1KYT0	0.43	0.006	0.54
Q4GZT3	0.23	0.004	0.24
Q6UX06	0.12	<0.0001	0.10
Q88YW7	0.21	0.23	0.23
Q8KCH7	0.16	0.05	0.15
Q8RY95	0.86	<0.0001	0.97
Q92CI0	0.46	0.002	0.81
Q9BE40	0.03	<0.0001	0.009
Q9HD89	0.19	0.15	0.19
Q9UGM3	0.82	0.002	0.95
A0R075	0.21	0.23	0.23
Q8CRH4	0.17	0.19	0.19
Q03AZ2	0.62	<0.0001	0.84
B9JXW5	0.17	0.19	0.19
C5DJ44	0.15	0.02	0.15
E8VHU4	0.17	0.19	0.19
O66405	0.17	0.19	0.19
P0DOX7	0.37	0.001	0.48
Q28641	0.0002	<0.0001	<0.0001
P52332	0.17	0.19	0.19
Q0BYP2	0.17	0.19	0.19
Q0C7P6	0.34	0.003	0.43
Q38914	0.17	0.19	0.19
Q3LHN2	0.17	0.19	0.19
Q3U3V8	0.18	<0.0001	0.20
Q4ZX12	0.17	0.19	0.19
Q5NXM9	0.17	0.19	0.19
Q92T74	0.17	0.19	0.19

Q9VB30	0.17	0.19	0.19
A7LV62	0.0003	0.29	0.28
Q5LB89	0.002	0.03	0.22
Q8A6B2	0.001	<0.0001	0.39
A7LTK9	0.02	0.38	0.84
Q8A9J2	0.007	0.28	0.33
A7LW10	0.008	0.29	0.64
A7LWC0	0.02	0.27	0.29
Q8A8R0	0.0001	0.28	0.28
A0A0C4DH68	0.003	0.49	0.27
P0DSN7	0.03	<0.0001	0.25
Q9NY84	0.002	0.58	0.40
A0A2Q2TTZ9	0.0003	0.38	0.29
P01602	<0.0001	0.47	0.23
P01877	0.002	0.58	0.17
P04406	0.002	<0.0001	0.75
H0Y9Q1	0.03	0.29	0.39
P80188	0.008	0.06	0.49
J3QKX5	0.004	0.07	0.30
Q8N944	0.008	0.38	0.38
A0A6Q8PGX3	0.01	0.84	0.13
A0A0J9YVZ3	0.04	0.67	0.28
P0DUB6	0.005	0.28	0.26
J3QSA3	<0.0001	0.09	0.64
A0A494C0P6	0.0003	0.08	0.43
P55259	0.006	0.007	0.98
Q08380	0.008	0.02	0.39
P35908	0.003	0.86	0.57
A0A087WYX0	0.007	0.28	0.72

A0A2U1B3U7	0.01	0.49	0.49
A7M4Q3	0.005	<0.0001	0.28
Q5L7N5	0.005	0.26	0.70
A0A0A0MRA3	0.006	0.27	0.73
Q9Y623	0.007	0.04	0.03
O14983	0.01	0.04	0.27
H0YKJ4	<0.0001	0.04	0.03
P06732	0.009	0.74	0.02
A0A1G6CH38	0.01	0.37	0.21
A0A380YV02	0.008	0.04	0.008
Q8ABD6	0.008	0.28	0.58
A0A0G2JMB2	0.01	<0.0001	0.83
A0A286YEY5	0.009	0.008	0.29
A0A2R8YEU4	0.004	0.27	0.29
Q5JXI8	<0.0001	0.49	0.23
C9J7T9	0.01	0.003	0.49
D6RD17	0.008	<0.0001	0.38
K7EP73	<0.0001	0.03	0.29
P01871	0.002	0.29	0.29
A0A1G6CRZ8	0.01	0.07	0.58
Q5NV56	0.004	0.002	0.25
P28838	0.006	0.25	0.49

Supplementary Table 10-11. P values of two-way repeated measures ANOVA of fecal proteomics after FDR correction.

Protein ID	Diet	BMI	Diet*BMI
P01602	0.02	0.52	0.58
J3QSA3	0.01	0.15	0.82
H0YKJ4	0.007	0.07	0.22
Q5JXI8	0.005	0.53	0.56
K7EP73	0.004	0.05	0.55
Q8A8R0	0.003	0.34	0.56
A8K7I4	0.005	0.01	0.97
Q28641	0.005	0.007	0.007
P0DOX2	0.006	0.007	0.02
P10246	0.006	0.0007	0.01
A7LV62	0.005	0.34	0.57
A0A2Q2TTZ9	0.005	0.42	0.56
A0A494C0P6	0.004	0.12	0.70
P12883	0.007	0.0007	0.93
P98088	0.02	0.007	0.005
Q8A6B2	0.02	0.007	0.65
P04406	0.02	0.007	0.87
P01871	0.02	0.34	0.54
P04264	0.02	0.003	0.72
Q5LB89	0.02	0.05	0.62
Q9NY84	0.02	0.61	0.65
P01877	0.02	0.61	0.70
P06702	0.03	0.003	0.93
A0A0C4DH68	0.02	0.53	0.60
P35908	0.02	0.86	0.78
P15085	0.03	0.0007	0.02

J3QKX5	0.03	0.12	0.54
A0A2R8YEU4	0.03	0.33	0.56
P0DUB6	0.03	0.33	0.58
A7M4Q3	0.03	0.007	0.58
Q5L7N5	0.03	0.33	0.83
D6RD17	0.03	0.007	0.66
A0A380YV02	0.03	0.07	0.17
Q8ABD6	0.03	0.34	0.78
P55259	0.04	0.02	0.98
Q08380	0.04	0.04	0.65
Q9Y623	0.04	0.08	0.23
Q5NV56	0.04	0.03	0.58
P06732	0.04	0.74	0.20
A0A0A0MRA3	0.04	0.33	0.85
Q8A9J2	0.04	0.34	0.34
A7LW10	0.04	0.34	0.81
O14983	0.04	0.07	0.59
C9J7T9	0.04	0.007	0.74
A6KXL2	0.04	0.02	0.08
A0A087WYX0	0.04	0.33	0.85
A0A0G2JMB2	0.04	0.007	0.93
A0A1G6CH38	0.04	0.42	0.61
P28838	0.04	0.57	0.75
Q9XSC6	0.04	0.005	0.07
P80188	0.04	0.09	0.74
Q8N944	0.04	0.42	0.65
A0A286YEY5	0.04	0.02	0.55
A0A1G6CRZ8	0.04	0.25	0.89
P01591	0.04	0.007	0.02

A0A6Q8PGX3	0.04	0.85	0.68
A0A2U1B3U7	0.05	0.53	0.74
P01833	0.07	0.007	0.72
A7LTK9	0.08	0.42	0.93
A7LWC0	0.08	0.34	0.56
P19961	0.09	0.007	0.61
Q9BE40	0.10	0.007	0.63
P12955	0.10	0.07	0.17
H0Y9Q1	0.10	0.34	0.66
P0DSN7	0.10	0.007	0.58
Q0VCY0	0.11	0.007	0.19
A6LFQ4	0.11	0.005	0.17
Q6GPI1	0.11	0.01	0.58
A0A0J9YVZ3	0.12	0.68	0.57
P14410	0.11	0.0006	0.21
Q5KR49	0.12	0.003	0.24
P05451	0.12	0.03	0.21
B3H1D9	0.13	0.06	0.22
P18600	0.13	0.0004	0.22
Q8A6N4	0.15	0.10	0.39
P06870	0.17	0.009	0.72
P15086	0.21	0.007	0.16
Q8A1A2	0.23	0.011	0.32
Q8A9M2	0.23	0.007	0.42
Q8AAW1	0.25	0.001	0.27
C4ZBL1	0.21	0.06	0.26
P04746	0.25	0.02	0.20
Q9BYE9	0.27	0.03	0.60
Q6UWV6	0.27	0.007	0.76

Q9TV62	0.26	0.007	0.39
Q9WUH4	0.27	0.15	0.55
Q5RDA4	0.27	0.007	0.78
Q6UX06	0.27	0.007	0.55
Q64MV4	0.27	0.007	0.58
P86210	0.28	0.007	0.51
P35527	0.28	0.04	0.59
A6L5A6	0.30	0.008	0.42
Q5R538	0.31	0.20	0.69
C5DJ44	0.31	0.04	0.71
P02788	0.32	0.007	0.72
Q8KCH7	0.32	0.08	0.71
Q8CRH4	0.35	0.27	0.70
B9JXW5	0.34	0.27	0.69
E8VHU4	0.34	0.26	0.68
O66405	0.34	0.26	0.66
P52332	0.33	0.26	0.65
Q0BYP2	0.33	0.26	0.64
Q38914	0.33	0.26	0.63
Q3LHN2	0.32	0.26	0.62
Q4ZX12	0.32	0.25	0.61
Q5NXM9	0.32	0.25	0.60
Q92T74	0.31	0.25	0.59
Q9VB30	0.31	0.25	0.58
Q02817	0.31	0.007	0.81
P84589	0.32	0.001	0.62
Q3U3V8	0.30	0.007	0.61
Q9HD89	0.32	0.22	0.72
P09093	0.35	0.007	0.63

Q47VD0	0.35	0.007	0.74
Q9NR71	0.34	0.21	0.72
C4ZF71	0.35	0.20	0.63
A6LGR3	0.36	0.29	0.63
P05208	0.35	0.29	0.62
P01721	0.35	0.29	0.61
P01887	0.35	0.29	0.60
Q88YW7	0.35	0.29	0.51
A0R075	0.34	0.28	0.58
C4Z1J4	0.34	0.25	0.57
Q4GZT3	0.35	0.009	0.57
A5A6M6	0.35	0.007	0.58
A2ASS6	0.35	0.11	0.70
Q9TSX8	0.35	0.0009	0.58
Q5NVH5	0.35	0.27	0.57
P16233	0.35	0.12	0.58
Q02897	0.35	0.17	0.70
Q86UP6	0.36	0.007	0.59
P08454	0.35	0.26	0.58
A9KQ65	0.38	0.06	0.57
Q1WUJ3	0.37	0.10	0.78
P15144	0.39	0.007	0.71
P00688	0.40	0.007	0.57
Q8A414	0.45	0.009	0.57
Q9GLN7	0.45	0.007	0.65
Q0C7P6	0.47	0.007	0.69
P09923	0.47	0.007	0.70
A6L792	0.48	0.09	0.69
B2UYT8	0.48	0.05	0.78

P06731	0.48	0.08	0.59
P02565	0.50	0.007	0.72
P0DOX7	0.49	0.003	0.74
O22317	0.49	0.007	0.82
Q5RE69	0.52	0.007	0.80
Q1KYT0	0.56	0.01	0.77
Q9BE39	0.57	0.26	0.73
Q9Y6R7	0.57	0.007	0.61
P02766	0.56	0.007	0.78
Q92CI0	0.59	0.005	0.93
B5YFN3	0.60	0.55	0.76
Q2RLT8	0.60	0.03	0.77
Q9UQQ1	0.61	0.32	0.78
P20758	0.61	0.007	0.80
O73860	0.61	0.02	0.78
Q13228	0.61	0.007	0.78
P07478	0.61	0.007	0.79
P01009	0.62	0.0007	0.78
P20111	0.61	0.60	0.78
P00761	0.62	0.0007	0.80
Q9H3R2	0.63	0.03	0.81
P10994	0.64	0.61	0.80
Q9CQY9	0.63	0.64	0.80
P08217	0.64	0.007	0.62
Q9PE76	0.65	0.67	0.82
P00813	0.65	0.67	0.81
P78010	0.64	0.66	0.81
Q03403	0.64	0.66	0.80
P02763	0.65	0.37	0.83

P0DOX7	0.66	0.007	0.74
P0DOY3	0.69	0.05	0.86
Q03AZ2	0.69	0.007	0.92
P08861	0.69	0.007	0.97
P01876	0.70	0.001	0.87
P0DOX8	0.72	0.007	0.92
P27487	0.75	0.007	0.92
P04054	0.75	0.0007	0.95
P48052	0.76	0.03	0.93
P02768	0.77	0.18	0.92
O43451	0.76	0.007	0.92
O43895	0.78	0.003	0.96
Q7MWI7	0.79	0.007	0.96
P01012	0.82	0.04	0.96
Q16820	0.85	0.25	0.97
Q9UGM3	0.85	0.005	0.97
P0DOX6	0.85	0.03	0.97
P13538	0.85	0.003	0.97
Q8RY95	0.88	0.40	0.98
P04118	0.88	0.40	0.98
P12821	0.90	0.003	0.98
P60052	0.99	0.0009	0.96

Supplementary Table 10-12 Pearson’s correlation analyses between fecal microbiota and metabolites.

	Glycocholic acid	hexacosanoyl carnitine	Aminoacetone	Phytosphingosine	Sphinganine	Methylimidazoleacetic acid	20-carboxy-leukotriene-B4	lysine	Sphingosine	Ubiquinol	Phylloquinone	20-Hydroxyleukotriene E4	Vitamin K1 epoxide	Sphingosine 1-phosphate	(4Z,7Z,10Z,13Z,16Z,19Z)-Docosahexaenoic acid	Chondroitin	3-Dehydrosphinganine	Sulfatide	4-Coumarate	24-oxo-1α,25-dihydroxyvitamin D3	13'-carboxy-γ-tocopherol	11H-14,15-EETA	Proline	Methionine	Aminoadipate	Hexadecanoic acid	Hypoxanthine	Deoxyguanosine	Deoxyuridine	Cytidine	Thymine	Nicotinic acid	Pyruvic acid	Phosphorylcholine	Thioctic acid amide	(S)-2-Acetolactate	Dihydrolipoamide	N-Acetyllactosamine
Bacteroides	0.15	-0.12	-0.18*	-0.40**	0.03	-0.15	-0.18*	0.09	0.03	-0.14	-0.02	-0.03	-0.14	0.05	0.32**	-0.12	0.05	0.07	0.10	-0.21*	0.07	0.26**	-0.08	-0.05	-0.18*	-0.12	0.21*	0.09	-0.04	-0.16	-0.04	-0.15	0.11	-0.22*	-0.16	-0.17	0.06	0.18*
Prevotella 9	-0.13	0.13	0.16	0.14	0.09	0.12	0.14	0.04	0.04	0.13	0.03	0.10	0.09	-0.16	-0.07	0.03	0.08	-0.19*	0.17	0.20*	-0.09	-0.08	0.11	0.09	0.04	0.09	-0.23*	-0.04	0.08	0.20*	0.04	0.15	-0.006	0.05	0	0.06	-0.09	0.04
Faecalibacterium	-0.01	0.07	-0.01	-0.20*	0	-0.21*	-0.01	0.004	0.08	0.03	-0.04	0.06	-0.04	-0.07	0.13	0.08	-0.04	-0.03	-0.27**	-0.01	-0.02	0.07	-0.08	-0.19*	-0.24**	-0.12	0.16	0.14	-0.01	-0.07	-0.25**	-0.25**	0.06	0.04	0.06	-0.19*	-0.05	0
Roseburia	0.05	0.04	0	0.04	0	-0.02	0.02	-0.02	0	-0.09	0.04	0.02	0	0.05	0.10	0.04	0.03	0.08	-0.19*	0.07	-0.10	0.12	-0.12	0.05	-0.005	0.08	0.15	0.06	-0.007	0.04	-0.13	-0.10	0.13	0.03	-0.05	0.12	-0.03	0.03
Lachnospira	0.04	-0.18*	-0.19*	-0.27**	-0.1	-0.30**	-0.15	-0.01	-0.15	-0.15	0.04	-0.1	-0.16	0.07	0.29**	-0.10	-0.04	0.08	-0.13	-0.1	0.03	0.17	-0.22*	-0.1	-0.09	0.02	0.27**	0.15	-0.10	-0.14	-0.29**	-0.18*	0.18*	-0.04	-0.13	0.004	0.05	0.16
Parabacteroides	0.18*	0.03	-0.08	-0.08	0.09	0.05	0.03	0.04	0.006	-0.005	0.12	0.04	0.10	-0.04	-0.13	0.004	-0.06	-0.13	-0.05	0.03	-0.006	-0.01	0.07	-0.02	-0.01	-0.06	0.08	0.11	0.03	0.08	0.02	-0.09	-0.03	-0.09	-0.1	-0.05	0.06	-0.04
Lachnoclostridium	0.05	0.11	0.005	-0.25*	0.22*	-0.26*	0.03	0.12	0.13	0.01	-0.07	0.12	0.08	-0.08	0.21*	0.25*	0.24*	-0.04	0.16	-0.07	-0.05	0.10	-0.06	-0.21*	-0.03	0.03	0.08	0.09	0.02	-0.04	0.08	-0.24*	-0.003	-0.05	-0.08	-0.17	-0.17	0.02

				*		*										*	*															*						
Ruminococcus 2	0.04	-0.05	-0.05	-0.08	-0.1	-0.02	-0.01	-0.02	-0.1	-0.11	0.06	-0.08	-0.08	0.01	-0.04	-0.10	-0.08	-0.03	-0.32**	0.09	-0.04	-0.02	0.04	0.007	-0.002	0.008	0.05	0.04	-0.03	0.05	-0.09	0.11	0.13	-0.03	-0.16	0.17	0.26**	0.03
Phascolarctobacterium	-0.11	-0.07	-0.11	0.03	-0.1	-0.03	-0.08	0.07	-0.03	0.05	-0.24**	-0.05	-0.11	0.09	0.15	-0.02	-0.03	0.21	0.06	0.10	0.31**	0.10	0.13	-0.06	-0.28**	-0.04	0.01	-0.15	0.06	0.05	0.04	-0.05	0.01	0.04	-0.02	-0.07	0.08	0.06
Prevotella 2	0.03	0.008	-0.15	0.14	-0.1	0.09	-0.09	0.01	-0.05	-0.02	-0.02	-0.12	-0.02	0.09	-0.13	-0.03	-0.07	0.07	-0.07	0.06	0.13	-0.1	-0.001	0.11	-0.02	0.01	0.05	-0.07	0.04	-0.03	0.01	0.12	-0.03	0.001	-0.01	0.11	0.19*	0.01
Agathobacter	-0.09	-0.05	-0.03	0.13	-0.1	-0.01	0.08	-0.03	0.04	0.08	-0.02	0.07	0.13	0.01	-0.06	0.09	-0.15	-0.05	-0.18*	0.11	-0.003	-0.003	-0.17	-0.13	0.04	0.001	0.10	-0.02	-0.24**	-0.12	-0.24**	-0.02	0.006	0.12	0.12	-0.02	0.17	-0.07
Alistipes	0.10	0.08	0.12	0.16	-0.1	0.07	0.08	-0.16	0.09	0	0.12	0.006	0.17	0.07	-0.27**	-0.03	-0.07	0.05	-0.21*	0.15	-0.02	-0.17	0.14	0.14	0.07	0.14	-0.11	-0.11	0.1	0.11	0.10	0.05	-0.1	0.03	0.03	0.26**	0.15	-0.24**
Alloprevotella	-0.18*	0.05	-0.03	0.20*	0	0.002	0.04	0.20*	-0.26**	0.11	0	-0.007	-0.02	0.02	-0.08	0.009	0	-0.002	0.11	0.1	0.10	0.05	0.009	-0.02	0.13	0.19*	-0.12	0.03	0.1	0.05	0.009	0.13	0.002	0.03	-0.04	0.02	0.01	-0.08
Fusobacterium	0.001	-0.06	-0.14	-0.23*	0.09	-0.12	-0.05	0.09	-0.05	0.03	-0.1	0.06	-0.13	-0.15	0.32**	0.12	0.14	-0.002	0.13	-0.08	0.03	0.24**	0.009	-0.29**	-0.28**	-0.16	0.15	0.17	0.01	-0.06	-0.02	-0.19*	-0.05	0.02	-0.08	-0.17	-0.18*	0.18*
Blautia	-0.09	0.16	0.12	-0.20*	0.14	-0.17	0.20*	0.10	0.20*	0.23**	-0.1	0.21*	0.14	-0.05	0.20*	0.33**	0.12	-0.06	-0.14	-0.01	-0.15	0.08	-0.17	-0.24**	-0.003	0.007	0.09	0	-0.11	-0.11	-0.21*	-0.19*	-0.08	0.26**	0.22*	-0.19*	-0.22*	-0.16
Dialister	0.04	0.26*	0.18*	-0.05	0.18*	0.08	0.12	-0.02	-0.03	-0.04	0.11	0.08	0.13	-0.14	-0.11	-0.02	0.08	-0.11	-0.06	-0.01	-0.24*	-0.11	-0.03	0.06	0.09	0.02	0.02	0.25*	0.14	0.04	0.05	-0.03	-0.04	-0.06	-0.03	0.10	-0.15	0.01

		*																			*							*										
Parasutterella	-0.01	0.04	-0.05	-0.02	0.09	-0.12	0.05	0.07	-0.08	-0.11	0.004	0.05	-0.01	0.09	0.02	0.10	0.01	0.03	-0.05	0	-0.03	0.04	-0.02	-0.12	0.07	-0.12	-0.09	-0.03	-0.15	-0.11	-0.06	0.03	0.02	0.15	-0.02	-0.007	0.05	-0.04
Muribaculaceae_norank	0.04	0.02	0.12	0.21*	-0.1	0.22*	0.02	-0.01	-0	0.06	0.06	-0.02	0.005	0.09	-0.28**	-0.06	-0.12	-0.07	0.08	0.17	-0.01	-0.07	0.14	0.08	0.07	0.005	0.01	-0.21*	0.02	-0.05	0.11	0.20*	0.07	-0.05	0.009	0.12	-0.03	0.07
Ruminococcaceae UCG-002	0.02	0.12	0.25**	0.48**	-0.1	0.31**	0.15	-0.1	0.06	-0.02	0.08	-0.002	0.25**	-0.05	-0.45**	0.02	-0.09	0.002	-0.02	0.23**	-0.02	-0.29**	0.19*	0.18*	0.18*	0.26**	-0.23**	-0.17	0.06	0.14	0.17	0.20*	-0.11	0.05	0.06	0.35**	0.08	-0.20*
Lachnospiraceae_uncultured	0.03	0.14	0	-0.14	0.25**	-0.14	0.18*	0.08	0.21*	0.10	-0.01	0.23**	0.18*	0.04	0.07	0.29**	0.18*	-0.08	0.08	0.14	-0.15	0.009	-0.04	-0.14	0.05	0.20*	0.03	-0.06	-0.07	0.12	-0.07	-0.16	-0.11	0.05	0.006	-0.08	-0.18*	-0.02
Megamonas	-0.09	0.03	-0.03	0.23	0	-0.06	0.007	0.07	-0.09	0.05	0.08	-0.07	0.01	-0.01	-0.06	0.005	0.04	0.11	-0.01	0.11	0.11	-0.05	-0.09	-0.09	-0.08	-0.05	-0.15	0.21*	0.02	-0.02	0.17	-0.04	-0.10	0.02	-0.01	0.03	-0.03	0.02
Escherichia-Shigella	0.001	0.14	0.09	0.12	0.22*	0.03	0.18*	-0.04	-0.06	0.01	-0.05	0.15	0	-0.08	0.028	0.27**	0.07	-0.01	-0.03	-0.16	-0.16	-0.04	-0.16	-0.15	0.04	0.06	-0.09	0.13	-0.03	0.02	0.03	-0.07	-0.16	0.10	0.12	-0.10	-.23**	-0.11
Sutterella	-0.04	-0	0.10	-0.04	-0.1	0.09	-0.02	-0.06	0.03	0.06	-0.01	-0.06	0.08	-0.08	-0.007	-0.09	0.03	0.02	-0.10	-0.06	0.05	0.007	0.08	0.09	-0.05	0.09	0.07	-0.03	0.11	0.06	0.02	-0.05	0.06	-0.14	-0.02	0.04	0.07	0.02
Megasphaera	-0.11	-0.07	0.09	-0.13	-0.1	-0.12	0.01	-0.07	0.02	-0.02	-0.12	0.005	-0.03	-0.16	0.21*	-0.02	0.17	0.07	-0.08	-0.13	0.05	0.14	-0.10	-0.05	-0.25**	0.06	-0.07	0.14	0.11	0.07	-0.05	-0.18*	0.14	-0.01	0.02	-0.03	0.08	0.001
Bifidobacterium	0.17	0.16	0.25**	-0.12	0.14	-0.07	0.17	-0.06	0.25**	-0.03	0.08	0.14	0.31**	-0.23*	-0.01	0.20*	0.19*	-0.20*	-0.14	-0.14	-0.23*	-0.03	-0.11	-0.11	0.004	0.007	0.06	0.04	-0.03	0.03	-0.23*	-0.23**	-0.04	0.03	0.24**	-0.09	-0.17	-0.19*
Subdoligranulum	0.12	0.06	0.11	0.29*	0.02	0.04	0.07	-0.04	-0.11	-0.12	0.14	0.004	0.10	-0.06	-0.18*	0.05	0.15	-0.05	-0.09	0.11	-0.07	-0.15	0.006	0.09	0.03	0.11	-0.07	0.10	0.19*	0.13	0.06	0.08	0.05	-0.05	-0.06	0.24*	0.01	-0.10

				*																															*		
Paraprevotella	0.02	0.05	0.05	0.13	0.01	-0.07	0.06	0.02	0.07	0.009	0.21*	0.03	0.03	0.02	-0.17	-0.007	-0.13	-0.04	-0.02	0.08	0.02	-0.01	0.13	0.05	0.02	0.11	-0.07	0.07	0.04	0.03	0.08	-0.001	-0.04	-0.02	-0.01	0.005	0.16	-0.18*
Ruminococcus 1	0.08	0.02	0.01	0.22*	-0.1	0.16	0.02	-0.05	0.07	-0.07	0.12	-0.03	0.21*	0.08	-0.27**	-0.09	-0.11	0.06	-0.09	0.17	0.04	-0.07	0.09	0.11	0.05	0.17	-0.06	-0.08	0.02	0.10	0.05	0.05	0.02	-0.07	-0.01	0.28**	0.04	-0.11
Haemophilus	-0.11	-0.02	-0.03	-0.26**	-0.1	-0.17*	-0.02	0.004	-0.17	-0.02	-0.03	-0.06	-0.11	-0.03	0.16	-0.06	0.006	0.04	-0.10	-0.04	-0.06	0.17	-0.09	-0.09	0.04	0.004	0.20*	0.07	-0.07	0.04	-0.24**	-0.04	0.02	0.05	-0.01	-0.01	0.03	0.002

Supplementary Method 1. Isolation of beef and chicken proteins.

Fat and connective tissue were manually removed from meat, and the muscles were finely chopped. The chopped meat was transferred into boiling bag and cooked in a 72 ℃ water bath until the center temperature of meat was 70 ℃. The cooked meat was chilled, freeze-dried, and ground into powder. Intramuscular fat was further removed using a mixed solvent of methylene chloride and methanol at a ratio of 2:1 (V/V), and organic solvent was removed in a fume hood.

Supplementary Method 2. In vitro static digestion.

Protein was mixed in SGF (containing 6.9 mM KCl, 0.9 mMKH_2PO_4, 25 mM $NaHCO_3$, 47.2 mM NaCl, 0.1 mM $MgCl_2$, 1 mM$(NH_4)_2CO_3$, and 15.6 mM HCl) to obtain a final concentration of 20 g/L, and the pH was adjusted by 1M hydrochloric acid to pH 3.0. Subsequently, pepsin was added to reach a final concentration of 2000 U/mL and gastric digestion was conducted at 37 ℃ and 200 rpm for 2 h. The gastric digestion was stopped by adding equal volume of SIF (containing 6.8 mM KCl, 0.8 mMKH_2PO_4, 85 mM $NaHCO_3$, 38.4 mM NaCl, 0.66 mM $MgCl_2$, and 8.4mM HCl). Pancreatin solution was added to reach a final concentration of 5 p-toluene-sulfonyl-L-arginine methyl ester (TEME) U/mL. The simulated intestinal digestion was conducted at 37 ℃ and 200 rpm for 2 h. Samples were heated at 95 ℃ for 5 min to inactivate pancreatin. All samples were stored at -20℃ prior to further analysis.
